# Cancer‐associated fibroblast migration in non‐small cell lung cancers is modulated by increased integrin α11 expression

**DOI:** 10.1002/1878-0261.12937

**Published:** 2021-03-25

**Authors:** Moe Iwai, Miniwan Tulafu, Shinsaku Togo, Hideya Kawaji, Kotaro Kadoya, Yukiko Namba, Jin Jin, Junko Watanabe, Takahiro Okabe, Moulid Hidayat, Issei Sumiyoshi, Masayoshi Itoh, Yu Koyama, Yasuhiko Ito, Akira Orimo, Kazuya Takamochi, Shiaki Oh, Kenji Suzuki, Yoshihide Hayashizaki, Koji Yoshida, Kazuhisa Takahashi

**Affiliations:** ^1^ Division of Respiratory Medicine Juntendo University Faculty of Medicine & Graduate School of Medicine Tokyo Japan; ^2^ Leading Center for the Development and Research of Cancer Medicine Juntendo University Tokyo Japan; ^3^ Tokyo Metropolitan Institute of Medical Science Setagaya‐ku Japan; ^4^ Preventive Medicine and Applied Genomics Unit RIKEN Center for Integrative Medical Sciences Yokohama Japan; ^5^ RIKEN Preventive Medicine and Diagnosis Innovation Program Saitama Japan; ^6^ Department of Respiratory and Critical Care Medicine National Center of Gerontology Beijing Hospital China; ^7^ Department of Pulmonology and Respiratory Medicine Universitas Indonesia Faculty of Medicine Jakarta Indonesia; ^8^ Departments of Molecular Pathogenesis Graduate School of Medicine Juntendo University Tokyo Japan; ^9^ Department of Oral Pathobiological Science and Surgery Tokyo Dental College Japan; ^10^ Department of General Thoracic Surgery Juntendo University School of Medicine Tokyo Japan; ^11^ Faculty of Biology‐Oriented Science and Technology Kindai University Wakayama Japan

**Keywords:** cancer‐associated fibroblast, cancer, stroma interaction, collagen type I, collagen type XI α1, integrin alpha‐11, non‐small‐cell lung cancer, transforming growth factor beta

## Abstract

Cancer‐associated fibroblasts (CAFs) regulate cancer progression through the modulation of extracellular matrix (ECM) and cancer cell adhesion. While undergoing a series of phenotypic changes, CAFs control cancer–stroma interactions through integrin receptor signaling. Here, we isolated CAFs from patients with non‐small‐cell lung cancer (NSCLC) and examined their gene expression profiles. We identified collagen type XI α1 (*COL11A1*), integrin α11 (*ITGA11*), and the *ITGA11* major ligand collagen type I α1 (*COL1A1*) among the 390 genes that were significantly enriched in NSCLC‐associated CAFs. Increased *ITGA11* expression in cancer stroma was correlated with a poor clinical outcome in patients with NSCLC. Increased expression of fibronectin and collagen type I induced *ITGA11* expression in CAFs. The cellular migration of CAFs toward collagen type I and fibronectin was promoted via ERK1/2 signaling, independently of the fibronectin receptor integrin α5β1. Additionally, ERK1/2 signaling induced *ITGA11* and *COL11A1* expression in cancer stroma. We, therefore, propose that targeting *ITGA11* and *COL11A1* expressing CAFs to block cancer–stroma interactions may serve as a novel, promising anti‐tumor strategy.

## Graphical abstract

Here, we analyzed gene expression profiles of non‐small‐cell lung cancer‐associated fibroblasts (CAFs). TGF‐β1 induced production of fibronectin and collagen type I in the cancer stroma, which next promoted the combined expression of integrin α11 and collagen type XI α1 in CAFs. Integrin α11^+^/collagen type XI α1^+^ CAFs relocalized within the cancer stroma in a fibronectin‐ and collagen type I‐dependent manner and promoted cancer cell migration.

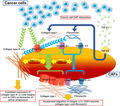

**Abbreviations**
CAF: cancer‐associated fibroblasts
CAGE: cap analysis of gene expression
COL11A1: collagen type XI α1
COL1A1: collagen type I α1
DEGs: differentially expressed genes
DMEM: Dulbecco’s modified Eagle medium
ECM: extracellular matrix
GO: gene ontology
HFL‐1: human fetal lung fibroblasts
HPF: high‐power fields
ITGA11: integrin α11
NSCLC: non‐small‐cell lung cancer
TGF‐β: transforming growth factor beta
α‐SMA: α‐smooth muscle actin

## 1 Introduction

Cancer‐associated fibroblasts (CAFs) constitute the key cellular components of cancer stroma in many solid cancer types and contain heterogeneous subpopulations that have distinct phenotypes and functions [1]. CAFs influence the major cancer hallmarks and promote malignant cancer cell conversion [2]. The continuous interactions of CAFs with cancer cells lead to altered fibroblast phenotypes with specific features, such as activated myofibroblasts. Furthermore, CAFs are responsible for excess extracellular matrix (ECM) deposition and potentially lead to cancer cell proliferation and migration to promote cancer development [3, 4, 5, 6]. Thus, targeting cancer‐associated stromal components, including CAFs, is essential for developing effective anti‐cancer therapies [7, 8, 9]. Although the CAF‐specific phenotype is directly related to accelerated cancer progression, its interaction with cancer cells has not been fully characterized. In this study, we focused on integrin α11 (ITGA11) and collagen type XI α1 (COL11A1) that are commonly overexpressed in non‐small‐cell lung cancer (NSCLC) tissues [10, 11]. We hypothesized that ITGA11 and COL11A1 may regulate altered CAF phenotypes and could play a role in cancer progression and associated poor prognoses. The pro‐fibrotic growth factor, transforming growth factor beta (TGF‐β) 1, accelerates trans‐differentiation to the activated myofibroblast and promotes ECM production and migration toward ECM signals, such as fibronectin [12, 13, 14, 15]. Furthermore, TGF‐β1‐stimulated collagen type I is a known ligand of ITGA11 in fibroblast [16]. TGF‐β1 stimulates both ITGA11 and COL11A1 in fibroblasts via the SMAD signaling pathway [17, 18]. Therefore, we analyzed whether ITGA11 and COL11A1 regulate CAF‐mediated bioactivity through fibronectin or collagen type I and could potentially serve as highly specific biomarkers for activated CAFs in NSCLC patients.

Integrins are heterodimeric transmembrane receptors composed of α and β subunits, and are involved in myofibroblast differentiation, cell adhesion, migration, and activation of the TGF‐β1 pathway in different fibrotic models [19, 20, 21]. The recent analysis of ITGA11, using highly selective mAbs, demonstrated expression of α11 in subsets of CAFs in various tumor types [22]. It will be interesting to determine which of the multiple collagen‐producing fibroblast subtypes in the lung [23] expresses ITGA11β1. However, ITGA11 expression was shown to be restricted to lung fibroblasts as a specific collagen receptor and was not detected in other stromal cell types [24]. While ITGA11 promotes myofibroblast differentiation and collagen reorganization, cancer–stromal ITGA11 expression has been found to be associated with cancer cell metastatic potential [17, 25, 26].

*COL11A1* encodes the collagen type XI α1 chain, a minor fibrillar collagen, which is a part of the common gene signatures associated with poor clinical outcomes [27, 28] and is known as a surrogate CAF biomarker in diverse cancer stroma [29, 30, 31, 32, 33].

In this study, we investigated whether CAFs have a unique fibroblast phenotype compared to common lung fibroblasts, using bioinformatic analyses on differentially expressed genes (DEGs). We introduced cap analysis of gene expression (CAGE) to analyze the comprehensive promoter activity in CAFs and identified potential mediators of CAF‐specific candidate genes that play a pivotal role in ECM‐mediated migratory capacity and ECM‐binding integrins through cancer cell–CAF interaction. We further examined the expression and localization of ITGA11 and COL11A1 in human CAFs and tumor tissues derived from NSCLC patients to validate ITGA11 and COL11A1 as specific cancer stroma biomarker.

## 2 Materials and methods

### 2.1 Patient samples and culture of lung fibroblasts

The Ethics Committee of Juntendo University School of Medicine approved this study (No. 18‐130). All subjects provided written informed consent to participate in this experimental study, according to our institutional guidelines. The study methodologies conformed to the standards set by the Declaration of Helsinki.

A total of 16 paired control (normal lung fibroblasts) and CAF samples were obtained from resected lung tissues of patients with pathologically diagnosed NSCLC from the Department of General Thoracic Surgery of Juntendo University School of Medicine. Primary lung fibroblasts were isolated from lung specimens as previously described [34]. Briefly, CAFs were isolated from the tumor tissue core. Paired normal lung fibroblasts (control) were obtained from the portions of lung parenchymal tissues as distal as possible from any tumor cells. Human primary fibroblasts derived from the tissue (referred to as ‘P0’) exhibited a typical spindle‐shaped fibroblast‐like morphology and were confirmed positive for vimentin and negative for cytokeratin staining. Passages 4 through 6 of primary lung fibroblasts were used for chemotaxis and ELISA to exclude the effect of differences in passage number and culture conditions.

The patients ranged in age from 44 to 79 years (63.9 ± 9.6); 13 of the patients were male, and three patients were female. There were 12 patients who smoked, and four who had never smoked. Histological differentiation was classed as high in three patients, moderate in five patients, and low in eight patients. The pathological stage was diagnosed as stage 1 in 11 patients, stage 2 in two patients, stage 3 in two patients, and stage 4 in one patient. The clinical pathology was adenocarcinoma in 11 patients and squamous cell carcinoma in five patients. Relapse occurred in five patients who had undergone surgery without preoperative chemotherapy or radiation (Table S1).

HFL‐1 human fetal lung fibroblasts (catalog no. CCL‐153) and A549 human lung adenocarcinoma (catalog no. CCL‐185) cell lines were purchased from American Type Culture Collection (Manassas, VA, USA); NIH 3T3 murine embryo fibroblast (catalog no. EC93061524‐F0), and BEAS‐2B human normal bronchial epithelium (catalog no. EC95102433) were purchased from European Collection of Authenticated Cell Cultures (Salisbury, UK). Cells were cultured in Dulbecco’s Modified Eagle Medium (DMEM; Wako Pure Chemical Industries, Osaka, Japan) supplemented with 10% FCS (Sigma‐Aldrich, St. Louis, MO, USA), 100 µg·mL^–1^ penicillin, 250 µg·mL^−1^ streptomycin, and 1 µg·mL^−1^ amphotericin B in a humidified atmosphere of 5% CO_2_. Sub‐confluent cells were removed from the dishes with 0.05% trypsin‐EDTA (Wako Pure Chemical Industries). TGF‐β1 was purchased from R&D Systems (Minneapolis, MN, USA). ERK inhibitor 3‐(2‐aminoethyl)‐5‐[(4‐ethoxyphenyl) methylene]‐2,4‐thiazolidinedione,monohydrochloride (catalog no. CAS1049738‐54‐6) was from Calbiochem (Dallas, TX, USA). Fibronectin (catalog no.11051407001) was from Roche (Mannheim, Germany). Collagen type I native protein derived from human placenta (NBP1‐97266) was from Novus Biologicals (Centennial, CO, USA). Recombinant human COL11A1 (catalog no.MBS718449_COA) was from MyBioSource (San Diego, CA, USA).

### 2.2 Fibroblast chemotaxis

Fibroblast‐mediated chemotaxis and A549‐mediated chemotaxis were measured using a Boyden blind‐well chamber (Neuro Probe, Gaithersburg, MD, USA) as previously described [35, 36]. In treatment experiments, TGF‐β1 (10 pm), recombinant human COL11A1, human fibronectin (20 µg·mL^−1^), or human collagen type I (1 µg·mL^−1^) was added to the cells of the upper chamber. Supernatant media from the cultured control fibroblasts or CAFs for A549‐mediated chemotaxis or human fibronectin (20 µg·mL^–1^), or human collagen type I (1 µg·mL^–1^) for fibroblast‐mediated chemotaxis was placed in the lower chamber as the chemoattractant. Chemotaxis was assessed by counting the number of cells in five high‐power fields (5HPF). Wells containing serum‐free DMEM served as negative controls.

### 2.3 Measurement of fibronectin, TGF‐β1, and collagen type XI α1 levels

Sub‐confluent lung fibroblasts grown in 6‐well plates were deprived of serum for 2 h and stimulated with or without 10 pm TGF‐β1. The supernatant from the cultured cells was harvested after 24 h and stored at −80 °C until analysis. Fibronectin, TGF‐β1, and COL11A1 production by the cells were determined using human fibronectin (R&D Systems), TGF‐β1 (R&D Systems), and COL11A1 (Abnova, Taipei, Taiwan) ELISA kits, respectively, according to the manufacturers' instructions.

### 2.4 Immunohistochemistry and scoring of histological staining in lung tissue

Lung samples were fixed in 10% neutral‐buffered formalin for 48 h, embedded in paraffin, and then cut in 4‐μm sections. Antigen retrieval was performed in EDTA/TRS buffer (pH 9.0; ITGA11) or in citrate buffer (pH 6.0; COL11A1). The sections were incubated with primary rabbit polyclonal antibody against ITGA11 (1 : 100 dilution, Ab198826; Abcam, Cambridge, UK) or COL11A1 (1 : 100 dilution, Ab64883, Cambridge, UK), followed by Dako REAL kit (K5007). Human liver cancer tissue was stained as a positive control for ITGA11 according to the manufacturer's datasheet. Human pancreatic ductal adenocarcinoma was used as the positive control for COL11A1, according to a previous report [29]. Rabbit IgG antibody (Ab172730) was used as a negative control (Fig. S1a).

The immunolabeling of markers was scored by two observers (ST and MI), without knowledge of the patient's clinical data, under a microscope at 100× magnification. Immunochemical staining for ITGA11 and COL11A1 was scored in a semi‐quantitative manner that reflected the staining intensity and percentage of area with stained cells. Staining intensity (I) was classified as 0 (no staining), +1 (weak staining), +2 (distinct staining), or +3 (strong staining). The percentage of positively stained cells (PC) was graded as 0 (0%), 1 (1–9%), 2 (10–49%), or 3 (≥ 50%). *H*‐scores were obtained as Σ (I × PC), where I and PC indicate the staining intensity and the percentage of cells stained at each intensity, respectively.

### 2.5 Western blot analysis

To standardize the culture conditions, HFL‐1 cells were cultured at a density of 0.5 × 10^5^ mL^−1^. The medium was changed to serum‐free DMEM for 24 h, and cells were treated with the designated concentration of TGF‐β1, CAS1049738‐54‐6, fibronectin, or collagen type I incubated in 37 °C.

For the conditioned media experiments, A549, BEAS‐2B, and HFL‐1 cells were cultured at a density of 1 × 10^5^ mL^−1^. The medium was harvested after changing to serum‐free DMEM for 48 h. HFL‐1 cells were cultured at a density of 0.5 × 10^5^ mL^−1^, and the harvested medium from the respective cell types was applied; cell lysates were prepared for western blotting after 0, 24, 48, and 72 h.

Primary antibodies against the following proteins were used for western blotting: ITGA11 (1 : 1000 dilution; Ab198826), COL11A1 (1 : 800 dilution; Ab64883), α‐smooth muscle actin; α‐SMA (1 : 1000 dilution; Sigma‐Aldrich; cat. no. A2547), fibronectin (1 : 1000 dilution; Enzo Life Sciences, Inc., Farmingdale, NY, USA; cat. no. BML‐FG6010‐0100), SMAD3 (1 : 800 dilution; Cell Signaling Technology, Beverly, MA, USA; cat. no. 9513), phospho‐SMAD3 (1 : 1000 dilution; Cell Signaling Technology; cat. no. 9520), p44/42 MAPK (ERK1/2) (1 : 2000 dilution; Cell Signaling Technology; cat. no. 4695), phospho‐p44/42 MAPK (ERK1/2) (Thr202/Tyr204) (1 : 2000 dilution; Cell Signaling Technology; cat. no. 4370), integrin α5 (A‐11) (1 : 500 dilution; Santa Cruz, Dallas, TX, USA; cat. no. sc‐166665), integrin β1 (A‐4) (1 : 500 dilution; Santa Cruz; cat. no. sc‐374429), and β‐actin (1 : 5000 dilution; FUJIFILM Wako Pure Chemical Corporation; cat. no. 281‐98721). Bound antibodies were visualized using peroxidase‐conjugated secondary antibodies and enhanced chemiluminescence with a LAS4000 image analyzer (GE Healthcare Bio‐Science AB, Uppsala, Sweden); band intensity was analyzed with an ImageQuant TL (GE Healthcare Bio‐Science AB).

### 2.6 Small interfering RNA‐mediated *ITGA11* knockdown

Small interfering RNAs (siRNAs) targeting *ITGA11* (Stealth RNAi HSS117658, HSS117660, HSS176942) were custom synthesized by Invitrogen (Life Technologies, Carlsbad, CA, USA). The sequences of the siRNA against *ITGA11* were as follows:

#1 Stealth RNAi HSS117658

sense: 5′‐GGGCCAGCAGAUAGGCUUACUUUAAAGU‐3′

anti‐sense: 5′‐AAAGUAAGAGCCUAUCUGCUGGCCC‐3′,

#2 Stealth RNAi,HSS117660,

sense: 5′‐GGCUUUCCAGAAGGGUGGAAGGAAA‐3′,

anti‐sense: 5′‐UUUCCUUCCACCCUUCUGGAAAGCC‐3′,

#3 Stealth RNAi HSS176942,

sense: 5′‐GGGCCUCCCUUCAGCUGCAUCUUCA‐3′,

anti‐sense: 5′‐UGAAGAUGCAGCUGAAGGGAGGCCC‐3′.

The siRNA negative control was purchased from Invitrogen (cat. no. #12935400, Invitrogen by Life Technologies). HFL‐1 cells were plated with 1 × 10^5^ mL^−1^ onto 60 mm dishes. Before transfection, the cells were grown to 50–80% confluence and medium was changed to serum‐free DMEM without antibiotics. The siRNAs were transfected into HFL‐1 cells using Lipofectamine reagent (Lipofectamine™ RNAiMAX; Lot no.2021708) at a concentration of 20 pmol·cm^−2^ dish. The transfection complex (siRNA and the transfection reagent mixture) were mixed in Opti‐MEM medium (Invitrogen, Life Technologies) and followed by incubation for 20 min at room temperature before added to cells. After 4–6 h, the cell culture medium was changed back to Opti‐MEM medium and incubated at 37 °C for 48 h. Transfected cells were then collected for western blotting and fibroblast chemotaxis as described above.

### 2.7 Retroviral vector construction and transfection

3.6 kb DNA fragment containing cDNA of human *ITGA11* with a C‐terminal DYKDDDDK (FLAG) was excised from pcDNA3.1(+)_*ITGA11*FLAG [37] by *EcoRI* and *XhoI* digestion and subsequently treated with Klenow fragment. The 3.6 kb DNA was ligated into *HincII*‐digested and CIAP (calf intestine alkaline phosphatase)‐treated pBABE‐puro (Addgene, Watertown, MA, USA). The constructed plasmid was transfected into HEK293T together with packaging and envelope vectors. The resulting viruses were infected into the target cells. After infection, transfected NIH 3T3 with either pBabe‐hTERT‐puro vector or PLKO‐1‐shRNA‐hygro vectors were cultured for 4–6 days and were selected in the presence of the appropriate antibiotic for each plasmid; puromycin (1 μg·mL^−1^) or hygromycin (50 μg·mL^−1^), respectively. Treated cells were also infected with nearly 90% infection efficiency by a PRRL‐GFP virus enriched by ultracentrifugation. Transfected cells were then collected after completed transfection for western blotting and chemotaxis as described above.

The retroviral vector encoding the full‐length transmembrane form of *ITGA11* cDNA was a gift from Kindai University Faculty of Biology‐Oriented Science and Technology Wakayama, Japan [37]. Additionally, a retroviral vector encoding the green florescence protein was used as a negative control [38].

### 2.8 CAGE analysis to identify candidate markers of CAFs

CAGE libraries were prepared using a previously described protocol [39]. Briefly, thawed human fibroblast cells were passaged every 3 days at a cell density of 1 × 10^5^ mL^−1^. The second passages were used to standardize the culturing condition. The cells were harvested, and double‐stranded RNA/cDNA was produced by reverse transcription from total RNA extracts using the SuperScript III kit (Life Technologies), purified, oxidized with sodium periodate to generate aldehydes from the diols of the ribose at the cap structure and 3ʹ‐end, and biotinylated with biotin hydrazide (Vector Laboratories, Burlingame, CA, USA). Single‐stranded cDNA was recovered after digestion of the single‐stranded RNA with RNase I (Promega, Madison, WI, USA) and ligated with 3ʹ‐end and 5ʹ‐end adaptors specific to the samples. Double‐stranded cDNAs were synthesized and mixed for sequencing in one lane of an Illumina HiSeq2500 sequencer (Illumina, San Diego, CA, USA). CAGE reads including a base ‘N’ or matching a ribosomal RNA sequence (U13369.1) identified by rRNAdust were excluded [40]. The CAGE reads were aligned to the reference genome (hg19) using Burrows‐Wheeler Aligner (version 0.7.10) [39], and comparison of quantified transcription between CAFs and control lung fibroblasts was indicated only with a high mapping quality of ≥ 20 using SAMtools (version 0.1.19) [41]. The robust peak set identified in the FANTOM5 project [42, 43] was used as a reference set for the transcription start sites; the number of mapped reads starting from these regions was used as the raw signal for promoter activities.

### 2.9 Graphing and statistical analysis

Comparisons between the control fibroblasts and CAFs were performed with paired Wilcoxon tests when paired samples within a group were available. Two‐tailed Mann–Whitney *U*‐test and unpaired Student's *t*‐tests were used for comparisons between the two unpaired groups. Bonferroni correction and Dunnett's test were used for the grouped data. Spearman was used for the correlation. For these comparisons, each subject was considered as an individual data point. Differences were considered statistically significant at *P* values < 0.05. Data were analyzed using prism 7 software (GraphPad Inc., San Diego, CA, USA). Results were presented as mean ± SD of at least three independent experiments to confirm the same tendency, and a representative picture was shown. For CAGE reads, expression (activity) levels of individual promoters for differential analyses were quantified as counts per million (CPM) after normalization by the relative log expression method [41] and subjected to differential analysis using edgeR (version 3.2.4) [44] in R/Bioconductor [45]. Gene ontology (GO) enrichment analysis was performed using david software (Laboratory of Immunopathogenesis and Bioinformatics, Frederick, MD, USA) [46].

## 3 Results

### 3.1 Promoter activity profiles in cancer‐associated fibroblasts and normal fibroblasts

**Fig. 1:**
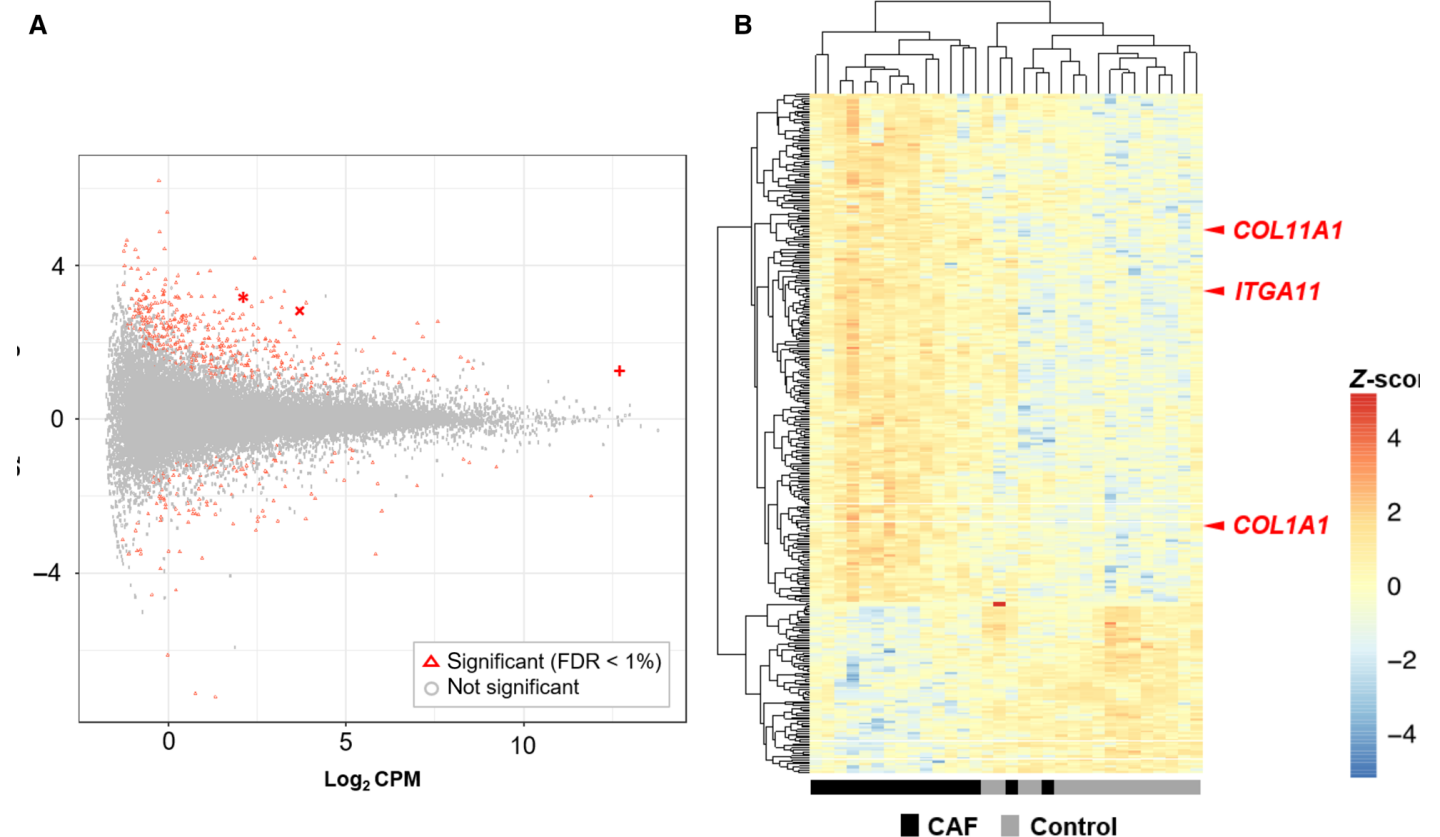
Promoter activity profiles in cancer‐associated fibroblasts and normal fibroblasts. (A) Average expression levels (*X*‐axis, CPM in log_2_ scale) and fold changes (*Y*‐axis, log_2_ scale) between CAFs and normal fibroblasts. Differentially expressed promoters with statistical significance (FDR < 1%) are shown in red. The most active promoters among *COL1A1*, *ITGA11,* and *COL11A1* are indicated by crossed dots; +, x, and asterisk;* respectively. (B) Heatmap demonstrating the individual promoter activity of the differentially expressed promoters (FDR < 1%); those with a certain level of expression (more than two CPM on average) and difference of expression (more than 2‐fold change) are shown. The hierarchical clustering across promoters and samples is based on Pearson's correlation coefficient with complete linkage method. (*n* = 16; pairs of control and corresponding CAF).

To demonstrate the quantitative profiles of genome‐wide CAF promoter activity, we analyzed 16 sample pairs of CAFs and their corresponding control fibroblasts using a CAGE protocol [39]. Statistically significant differentially expressed promoters (false discovery rate, FDR < 1%) (Fig. 1A) were identified in CAFs from NSCLC tissues, and 390 and 121 promoters were upregulated and downregulated, respectively (Table S2). Three distinct phenotypes between CAFs and normal fibroblasts were demonstrated by heat map analysis (Fig. 1B). We identified DEGs that altered promoter activity in CAFs vs. normal fibroblasts, including the previously reported CAF‐specific markers, *POSTN* and *PDPN* [9]. We then focused on three enriched gene candidates as CAF‐specific markers, *COL11A1*, *ITGA11,* and its ligand *COL1A1* (Fig. 1). *COL11A1* and *ITGA11* enriched four isotype promoters, each, and *COL1A1* enriched nine isotype promoters (Table S2). Two GO terms, ECM organization and cell adhesion, were identified in CAFs including *ITGA11* and *COL1A1*. ECM organization also included *COL11A1* (Table S3). Cell adhesion is one of the most significant GO terms for biological processes including the integrin‐mediated fibrogenic process, suggesting that ITGA11‐mediated interaction with the ECM promotes bioactivity of CAFs [47].

### 3.2 Immunohistochemical expression of integrin α11 and collagen type XI α1 in cancer stroma

**Fig. 2:**
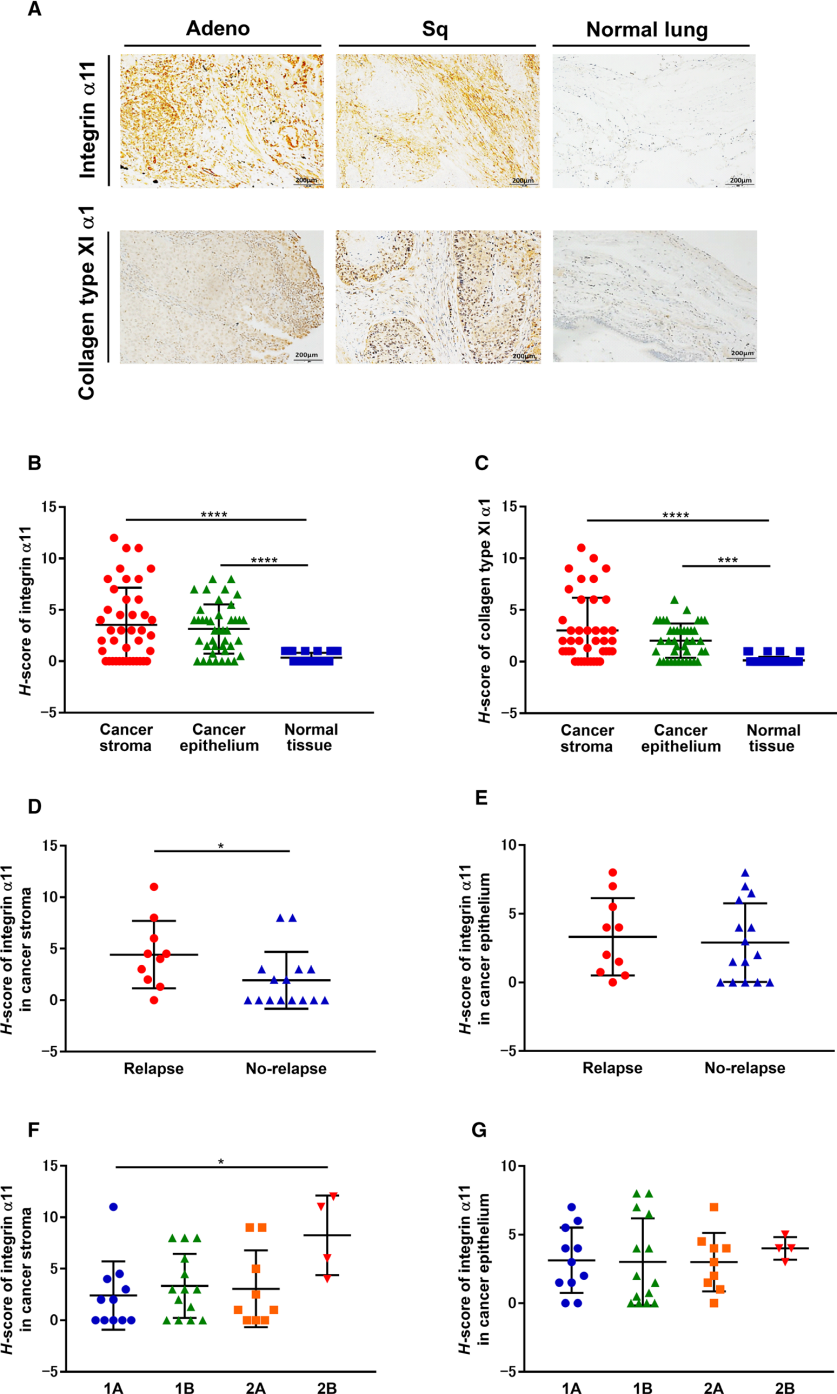
Immunohistochemical expression of ITGA11 and COL11A1 in cancer stroma (A) Immunohistochemical staining of human NSCLC tissues; representative sections of Adeno, Sq, and normal lung with ITGA11 and COL11A1 expressions are shown. Original magnification: 100×; Scale bars: 200 μm. (B) ITGA11 and (C) COL11A1 expression levels in each subject. (B, C; *n* = 41, one‐way ANOVA) Association between the recurrence and ITGA11 expression in (D) cancer stroma and (E) cancer epithelium. (D, E; *n* = 25, Mann–Whitney) Association between the pathological staging and ITGA11 expression in (F) cancer stroma and (G) cancer epithelium. (F, G; *n* = 38, one‐way ANOVA) Vertical axis: *H*‐score values (see Section 2.4). Each symbol represents one patient. The values represent the mean ± SD. *P < 0.05, ***P < 0.001, ****P < 0.0001. Adeno, Adenocarcinoma; Sq, squamous cell carcinoma.

Representative tumor sections showed higher ITGA11 and COL11A1 expressions in intratumoral cancer epithelium and cancer stroma compared to normal lung tissue (Fig. 2A). Appropriate positive and negative controls were stained according to the manufacturers' protocol and a previous report (Fig. S1a) [29].

Intratumoral expression levels (*H*‐score) of ITGA11 and COL11A1 in cancer epithelium and cancer stroma were significantly higher than those in normal lung tissue (Fig. 2B,C). Furthermore, ITGA11 expression in cancer stroma was closely correlated to the expression of COL11A1 in cancer stroma, but not to that in cancer epithelium (Table 1: *P* = 0.0006). In addition, the *H*‐score of ITGA11 in the cancer stroma was significantly higher in the relapse group than that in the nonrelapsed group following surgery (Fig. 2D), however, not in the cancer epithelium (Fig. 2E). We also analyzed the *H*‐score of ITGA11 in the cancer stroma and cancer epithelium at different pathological stages. In the cancer stroma, the *H*‐score of ITGA11 at stage 2B was significantly higher than that at stage 1A (Fig. 2F). However, the *H*‐score of ITGA11 in cancer epithelium was not associated with the pathological stage (Fig. 2G). In contrast, the *H*‐score of COL11A1 was not associated with recurrence or pathological stage (Fig. S2a–d). Thus, ITGA11 expression level in the cancer stroma involving CAFs serves as the crucial surrogate biomarker rather than that in the cancer epithelium itself.

**Table 1:** Correlation between ITGA11 and COL11A1 expression levels in cancer‐associated fibroblasts. *r*, correlation coefficient.

Measurement		*P*‐value	Spearman : *r*
Intratumoral immunostaining (*H*‐score)			
Cancer epithelium	Cancer stroma		
ITGA11	ITGA11	0.7176	−0.05823
Collagen type XI α1	Collagen type XI α1	0.7984	0.04114
ITGA11	Collagen type XI α1	0.3288	0.1564
Collagen type XI α1	ITGA11	0.332	−0.1554
Cancer stroma	Cancer stroma		
ITGA11	Collagen type XI α1	0.0006*	0.511
Cancer epithelium	Cancer epithelium		
ITGA11	Collagen type XI α1	0.252	0.183
Immunoblotting of CAFs			
ITGA11	Collagen type XI α1	0.1289	0.3971

* *P* < 0.001.

### 3.3 Cancer‐associated fibroblast migration and expression of integrin α11, integrin α5, integrin β1, and collagen type XI α1

**Fig. 3:**
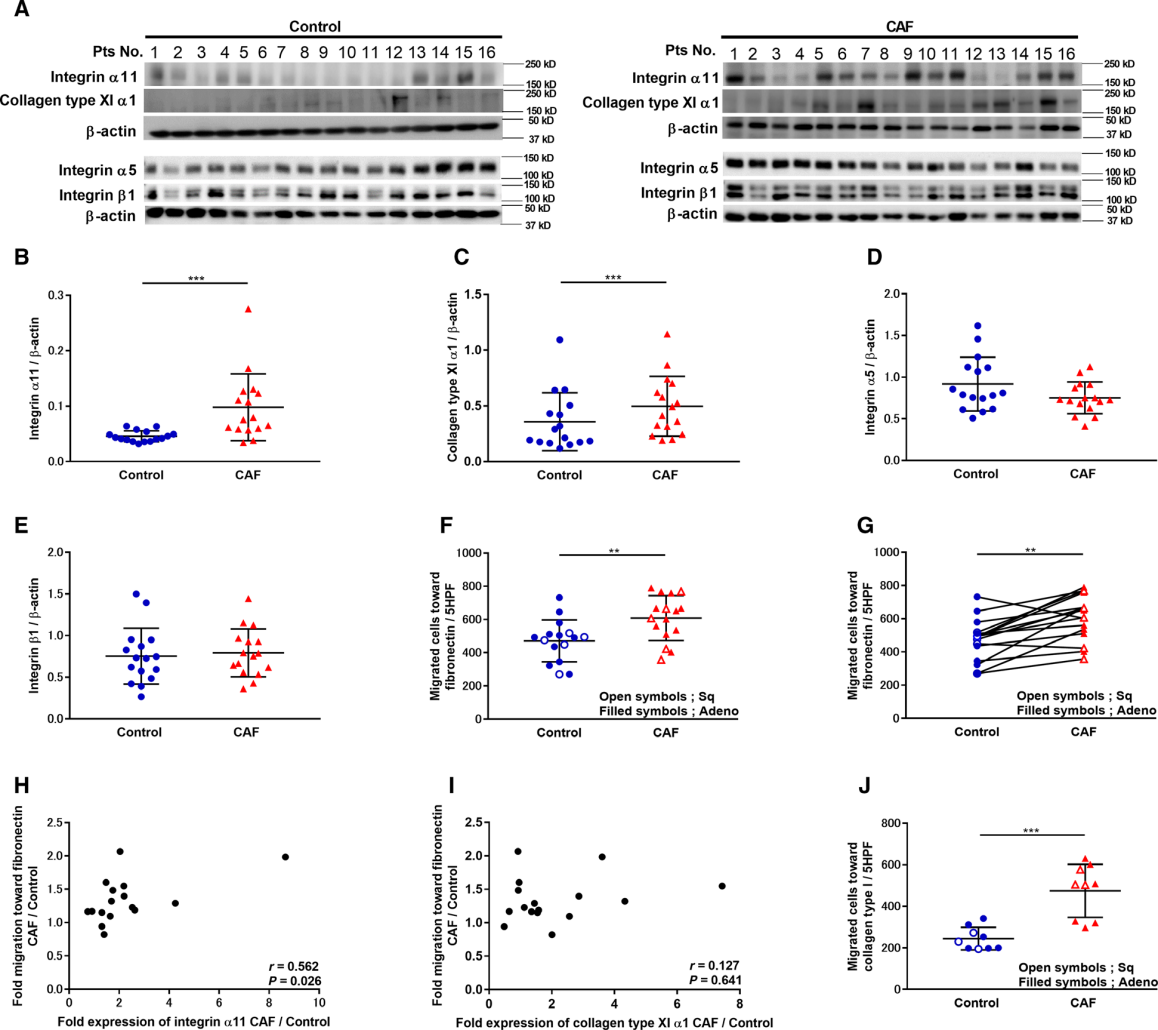
Cancer‐associated fibroblast migration and expression of ITGA11, integrin α5, integrin β1, and COL11A1. Sub‐confluent fibroblasts from 16 pairs of control fibroblasts and CAFs were cultured, and (A) proteins were extracted and analyzed by western blot to detect (B) ITGA11, (C) COL11A1, (D) integrin α5, and (E) integrin β1 expression. (B‐E; *n* = 16, Wilcoxon) Vertical axis: expression of protein normalized to β‐actin. (F) After sub‐confluent fibroblasts, from 16 pairs of control fibroblasts and CAFs, were cultured, chemotactic activity toward fibronectin (20 µg·mL^−1^) was measured. (F; *n* = 16, Mann–Whitney) Vertical axis: number of migrated cells per 5HPF. Filled symbols represent lung adenocarcinoma. Open symbols represent squamous cell lung cancer. (G) Comparison of CAFs and control fibroblasts from the same donor. (G; *n* = 16, Wilcoxon) Vertical axis: number of migrated cells per 5HPF. Filled symbols represent lung adenocarcinoma. Open symbols represent squamous cell lung cancer. (H) Relationship between fold CAF migration toward fibronectin (20 µg·mL^−1^) divided by corresponding control fibroblast migration and fold CAF expression of ITGA11 divided by corresponding control fibroblast. Vertical axis: fold CAF migration compared to control fibroblasts. Horizontal axis: fold ITGA11 expression in CAFs compared to control fibroblasts. (H; *n* = 16, Spearman) (I) Relationship between fold CAF migration toward fibronectin (20 µg·mL^−1^) divided by corresponding control fibroblast migration, and fold CAF expression of COL11A1 divided by corresponding control fibroblast. Vertical axis: fold CAF migration compared to control fibroblasts. Horizontal axis: fold COL11A1 expression in CAFs compared to control fibroblasts. (I; *n* = 16, Spearman) (J) After sub‐confluent patients’ control fibroblasts and CAFs were cultured, migration activity toward collagen type I (1 µg·mL^−1^) was measured. (J; *n* = 9, Mann–Whitney) Vertical axis: number of migrated cells per 5HPF. Filled symbols represent lung adenocarcinoma. Open symbols represent squamous cell lung cancer. Each symbol represents one patient. The values represent the mean ± SD. Pts: patients. **P < 0.01, ***P < 0.001.

Since *ITGA11* and *COL11A1* gene expression were upregulated in CAFs compared to the control fibroblasts, the protein expression level of ITGA11, COL11A1, and the associated protein expression levels of representative fibronectin receptors were assessed using western blotting (Fig. 3A, Fig S1b). CAFs expressed higher levels of ITGA11 (Fig. 3B: *P* = 0.0002) and COL11A1 (Fig. 3C: *P* = 0.0002) compared to control fibroblasts. Meanwhile, integrin α5 and integrin β1 expression levels of CAFs did not differ from that of control fibroblasts (Fig. 3D: *P* = 0.1167, Fig. 3E: *P* = 0.9399).

Next, the altered functional phenotypes of CAFs compared to control fibroblasts were examined by observing fibroblast‐mediated migration toward fibronectin. Migration ability toward fibronectin was higher in CAFs than in control fibroblasts (Fig. 3F,G: controls: 471.2 ± 121.7 cells per five HPF, CAFs: 608.5 ± 131.2 cells per five HPF). The result was significant when two different statistical comparison methods were used, that is, by separating the two groups between the control fibroblasts and CAFs (*P* = 0.005, Fig. 3F), and by using the CAFs‐corresponding control fibroblasts in the same donor as the paired analysis (*P* = 0.0013, Fig. 3G), although the fibronectin receptor expression levels were similar in both groups.

To validate the role of ITGA11 and COL11A1 in CAF migration, we investigated whether the expression levels of ITGA11 and COL11A1 in CAFs were related to their migration. CAF migration positively correlated with ITGA11 expression levels (Fig. 3H: *r* = 0.562, *P* = 0.026), but not with the expression levels of COL11A1 (Fig. 3I: *r* = 0.127, *P* = 0.641). Released COL11A1 in harvested media from CAFs and control fibroblasts was measured using ELISA, and the effect of recombinant human COL11A1 on HFL‐1 migration was investigated. CAFs released significantly higher levels of COL11A1 compared to the control fibroblasts (Fig. S3a: *P* = 0.0056); however, COL11A1 did not directly stimulate HFL‐1‐mediated migration toward fibronectin (Fig. S3b).

Since collagen type I is known to be the direct ligand of ITGA11, which is overexpressed in CAFs, we also examined the effect of collagen type I on CAF migration. Migration ability toward collagen type I was higher in CAFs than in control fibroblasts (Fig. 3J: controls: 244.6 ± 51.4 cells per five HPF, CAFs: 474.4 ± 120.4 cells per five HPF).

### 3.4 Response of cancer‐associated fibroblasts to TGF‐β1 and fibronectin

**Fig. 4:**
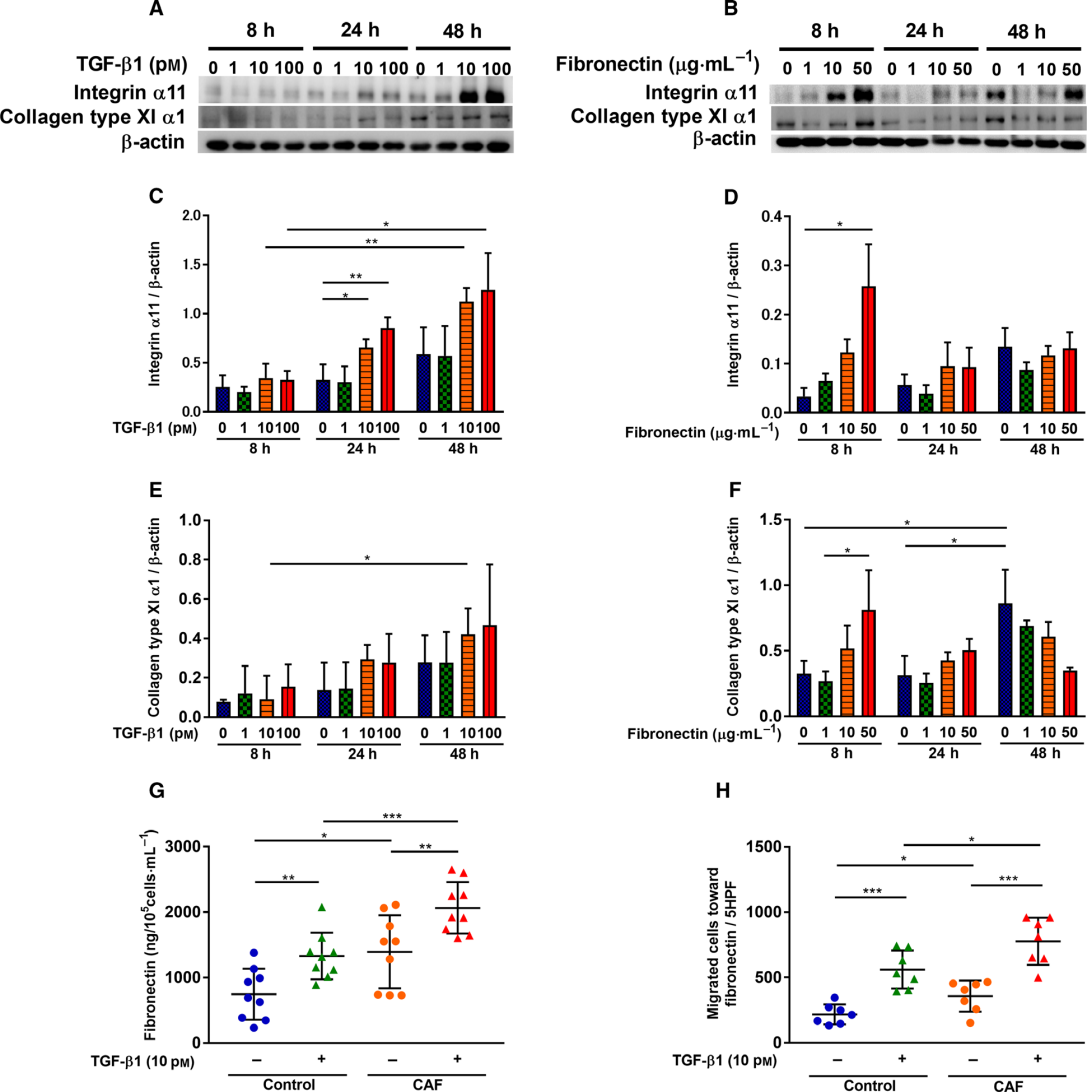
Response of cancer‐associated fibroblasts to TGF‐β1 and fibronectin. Sub‐confluent HFL‐1 were cultured and treated over different times and with different doses of (A) TGF‐β1 and (B) fibronectin. Proteins were extracted and subjected to western blot analysis to detect the time course of (C, D) ITGA11 and (E, F) COL11A1 expression level. (C‐F; *n* = 3, unpaired Student's *t*‐test) Vertical axis: expression of protein normalized to β‐actin. (G) Effect of TGF‐β1 on CAF fibronectin production compared to control fibroblasts. (G; *n* = 9, unpaired Student's *t*‐test) Vertical axis: fibronectin production expressed as the amount per 10^5^ cells mL^−1^. (H) Effect of TGF‐β1 in CAF migration toward fibronectin (20 µg·mL^−1^) compared to control fibroblasts. (H; *n* = 7, unpaired Student's *t*‐test) Vertical axis: number of migrated cells per 5HPF. Each symbol represents one patient. The values represent the mean ± SD. *P < 0.05, **P < 0.01, ***P < 0.001.

CAFs express the activated myofibroblast marker α‐SMA which is generally induced by TGF‐β1 stimulation [9]. Since TGF‐β1 stimulates α‐SMA production and fibronectin release, and is known as the autocrine or paracrine mediator of lung fibroblast‐dependent migration [48, 49], we assessed whether TGF‐β1 (Fig. 4A) and fibronectin (Fig. 4B) change the phenotype of HFL‐1 fibroblasts. More than 10 pm of TGF‐β1 significantly stimulated ITGA11 expression after 24 h (Fig. 4C), and fibronectin rapidly stimulated ITGA11 expression after 8 h (Fig. 4D). Collagen type XI α1 showed a similar tendency to ITGA11 (Fig. 4E,F). Next, we assessed whether the response to TGF‐β1 on fibronectin release and migration of CAFs was altered. Fibronectin release from CAFs (Fig. 4G: *P* = 0.0007) and migration of CAFs (Fig. 4H: *P* = 0.0295) were significantly higher than those of the control fibroblasts. Meanwhile, TGF‐β1 significantly stimulated fibronectin release (control fibroblasts: *P* = 0.0044, CAFs: *P* = 0.0095) and migration toward fibronectin (control fibroblasts: *P* = 0.0001, CAFs: *P* = 0.0002) in both groups, although the changes in the relative increase of fibronectin release and migration following TGF‐β1 treatment between groups were the same (Fig. 4G,H). Therefore, ITGA11 and COL11A1 expression levels induced by TGF‐β1 in CAFs altered their character at the point of fibronectin release.

The response to addition of exogenous TGF‐β1 between groups did not differ, yet an enhanced migration ability was observed in CAFs (Fig. 3F,G); therefore, we measured the endogenous TGF‐β1 release. The relative production of TGF‐β1 did not differ between the groups (Fig. S4), suggesting that the activated bioactivities of CAFs were partially independent on the TGF‐β1‐SMAD3 pathway [48].

### 3.5 Collagen type I‐mediated integrin subunit regulation and fibroblast migration

**Fig. 5:**
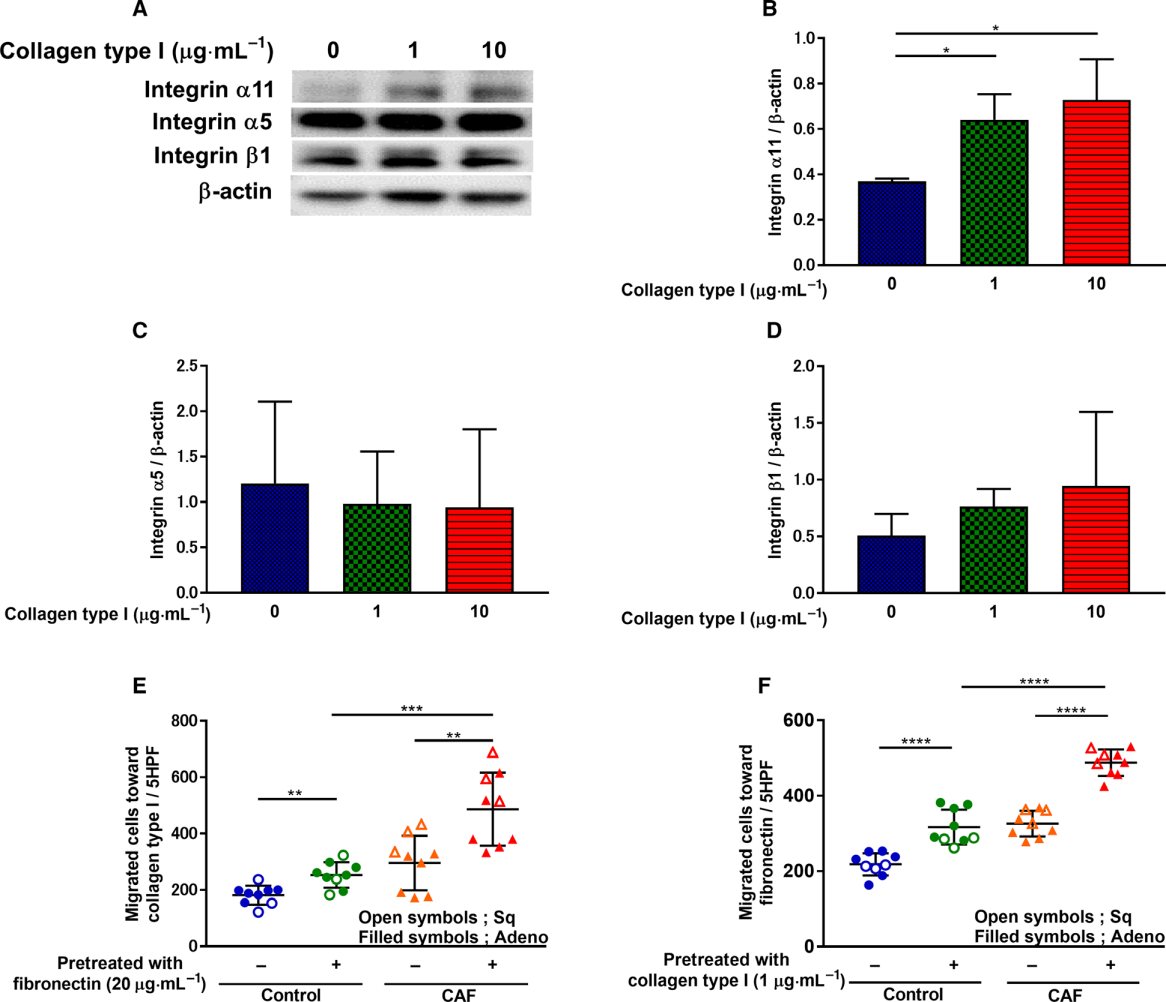
Collagen type I‐mediated integrin subunit regulation and fibroblast migration. After HFL‐1 were cultured and incubated with different doses of human collagen type I for 48 h, (A) proteins were extracted and subjected to western blot analysis to detect (B) ITGA11, (C) integrin α5, and (D) integrin β1 expression levels. (B, C, D; *n* ≥ 3, unpaired Student's *t*‐test) Vertical axis: expression of protein normalized to β‐actin. (E) After preincubation with human fibronectin (20 µg·mL^−1^) for 8 h, CAFs‐ and control fibroblasts‐mediated migration toward collagen type I (1 µg·mL^−1^) was measured. Vertical axis: number of migrated cells per 5HPF. (F) After preincubation with collagen type I (1 µg·mL^−1^) for 48 h, CAFs‐ and control fibroblasts‐mediated migration toward fibronectin (20 µg·mL^−1^) was measured. (E, F; *n* = 9, unpaired Student's *t*‐test) Vertical axis: number of migrated cells per 5HPF. Each symbol represents one patient. Filled symbols represent lung adenocarcinoma. Open symbols represent squamous cell lung cancer. The values represent the mean ± SD. *P < 0.05 **P < 0.01, ***P < 0.001, ****P < 0.0001.

To further elucidate the role of ITGA11‐mediated migration, we examined the effect of collagen type I on ITGA11, integrin α5, and integrin β1 expressions (Fig. 5A). The expression of ITGA11 was enhanced by collagen type I after 48 h treatment in a dose‐dependent manner (Fig. 5B); meanwhile, the expression of integrin subunits α5 and β1 did not change (Fig. 5C,D). Since fibronectin stimulated ITGA11 expression (Fig. 4D), we investigated the effect of 8 h pretreatment with fibronectin (20 µg·mL^−1^) on fibroblast migration toward collagen type I. The fibronectin pretreatment significantly stimulated HFL‐1 migration (Fig. S5) and enhanced CAF migration as compared to control fibroblasts toward collagen type I (Fig. 5E). Since collagen type I also stimulated ITGA11 (Fig. 5B), we investigated the effect of 48 h pretreatment with collagen type I (1 µg·mL^−1^) on migration toward fibronectin. Pretreatment with collagen type I (1 µg·mL^−1^) significantly stimulated HFL‐1 migration (Fig. S5) and enhanced CAF migration as compared to control fibroblasts toward fibronectin (Fig. 5F). These data suggest both fibronectin and collagen type I may play a role as a chemoattractant in accelerating ITGA11‐mediated CAF migration.

### 3.6 Regulation of ERK1/2 signal activation in cancer‐associated fibroblasts

**Fig. 6:**
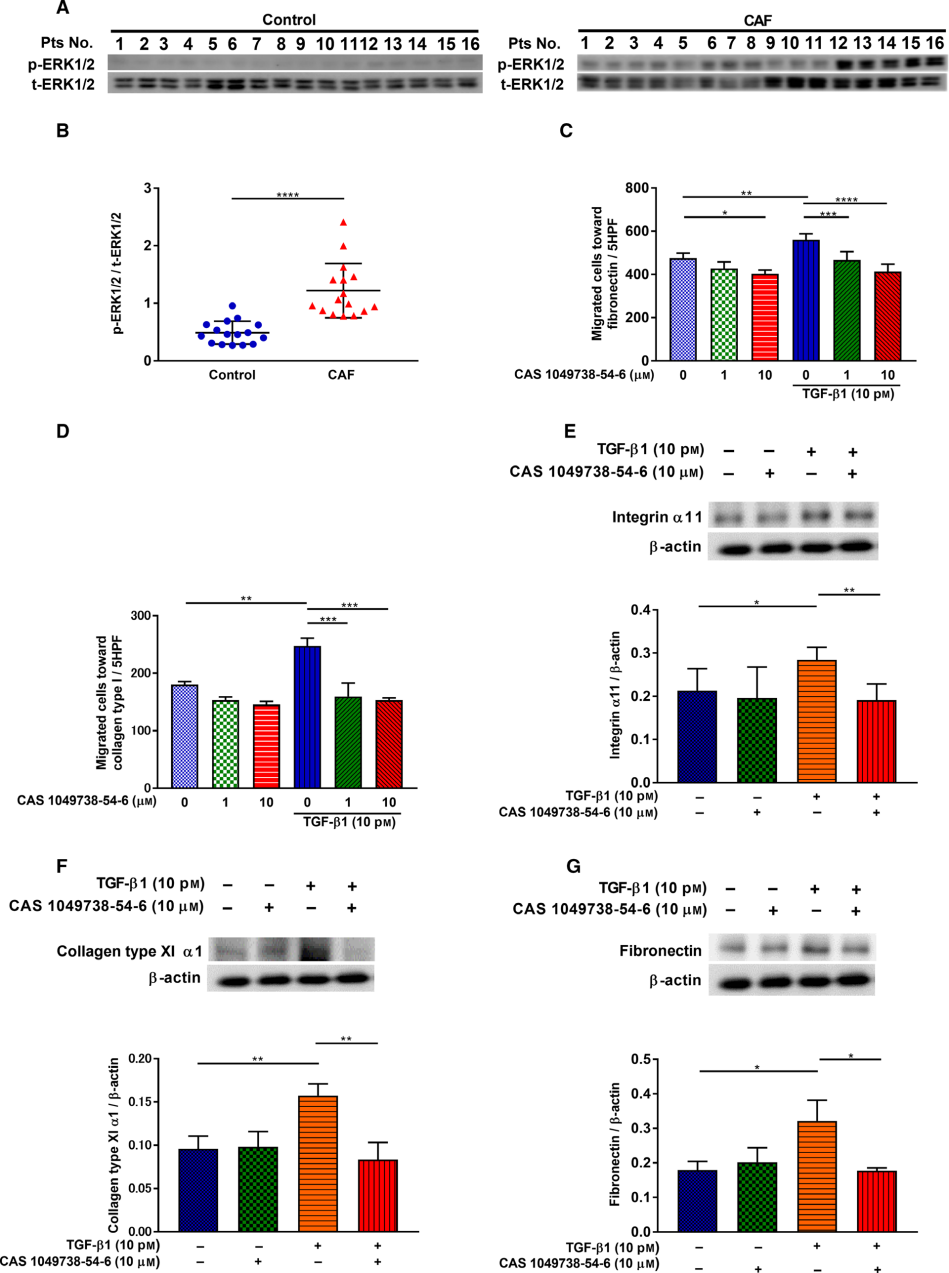
Regulation of ERK1/2 signal activation in cancer‐associated fibroblasts. (A) Sub‐confluent fibroblasts from 16 pairs of control fibroblasts and CAFs were cultured, and proteins were extracted and subjected to western blot analysis to detect (B) p‐ERK1/2 expression levels. Vertical axis: expression of protein normalized to t‐ERK1/2. (B; *n* = 16, Wilcoxon) (C) Effect of various concentrations of ERK inhibitor CAS 1049738‐54‐6, with or without TGF‐β1 (10 pm), on HFL‐1 cells was assessed with migration assay toward fibronectin (20 µg·mL^−1^). (C; *n* = 3, one‐way ANOVA) Vertical axis: number of migrated cells per 5HPF. (D) Effect of various concentrations of CAS 1049738‐54‐6, with or without TGF‐β1 (10 pm), on HFL‐1 cells was assessed with migration assay toward collagen type I (1 µg·mL^−1^). (D; *n* = 3, one‐way ANOVA) Vertical axis: number of migrated cells per 5HPF. Sub‐confluent HFL‐1 cells were cultured and treated with or without TGF‐β1 (10 pm), and CAS 1049738‐54‐6 (10 μm) for 24 h. Proteins were extracted and subjected to western blot analysis to detect the (E) ITGA11, (F) COL11A1, and (G) fibronectin expression levels. (E, F, G; *n* ≥ 3, unpaired Student's *t*‐test) Vertical axis: expression of protein normalized to β‐actin. Each symbol represents one patient. The values represent the mean ± SD. Pts: patients. *P < 0.05, **P < 0.01, ***P < 0.001, ****P < 0.0001.

Blockade of *ITGA11* gene expression reduces ERK activation in cancer cells [25, 50]; therefore, we examined the phosphorylation status of ERK1/2 in CAFs compared to control fibroblasts (Fig. 6A). The relative increase in p‐ERK1/2 was significantly higher in CAFs than in control fibroblasts (Fig. 6B). Furthermore, we investigated whether the ERK1/2 signal was related to the increased level of CAF migration. Treatment with ERK inhibitor, CAS 1049738‐54‐6, suppressed HFL‐1 migration toward fibronectin and collagen type I (Fig. 6C,D). The inhibitory effect of the CAS 1049738‐54‐6 on TGF‐β1‐stimulated HFL‐1 migration toward fibronectin was further suppressed as inhibitor concentration increased. These results showed that the activated bioactivities of CAF migration involved the ERK1/2 signaling pathway. To elucidate the role of ERK1/2 signaling in ITGA11, COL11A1 and fibronectin regulation, ITGA11, COL11A1, and fibronectin expression levels in HFL‐1 were measured. TGF‐β1‐induced ITGA11, COL11A1, and fibronectin expression were significantly suppressed by CAS 1049738‐54‐6 (10 μm) (Fig. 6E‐G).

### 3.7 Phenotypic alteration of lung fibroblasts via fibroblast–lung cancer cell interaction

**Fig. 7:**
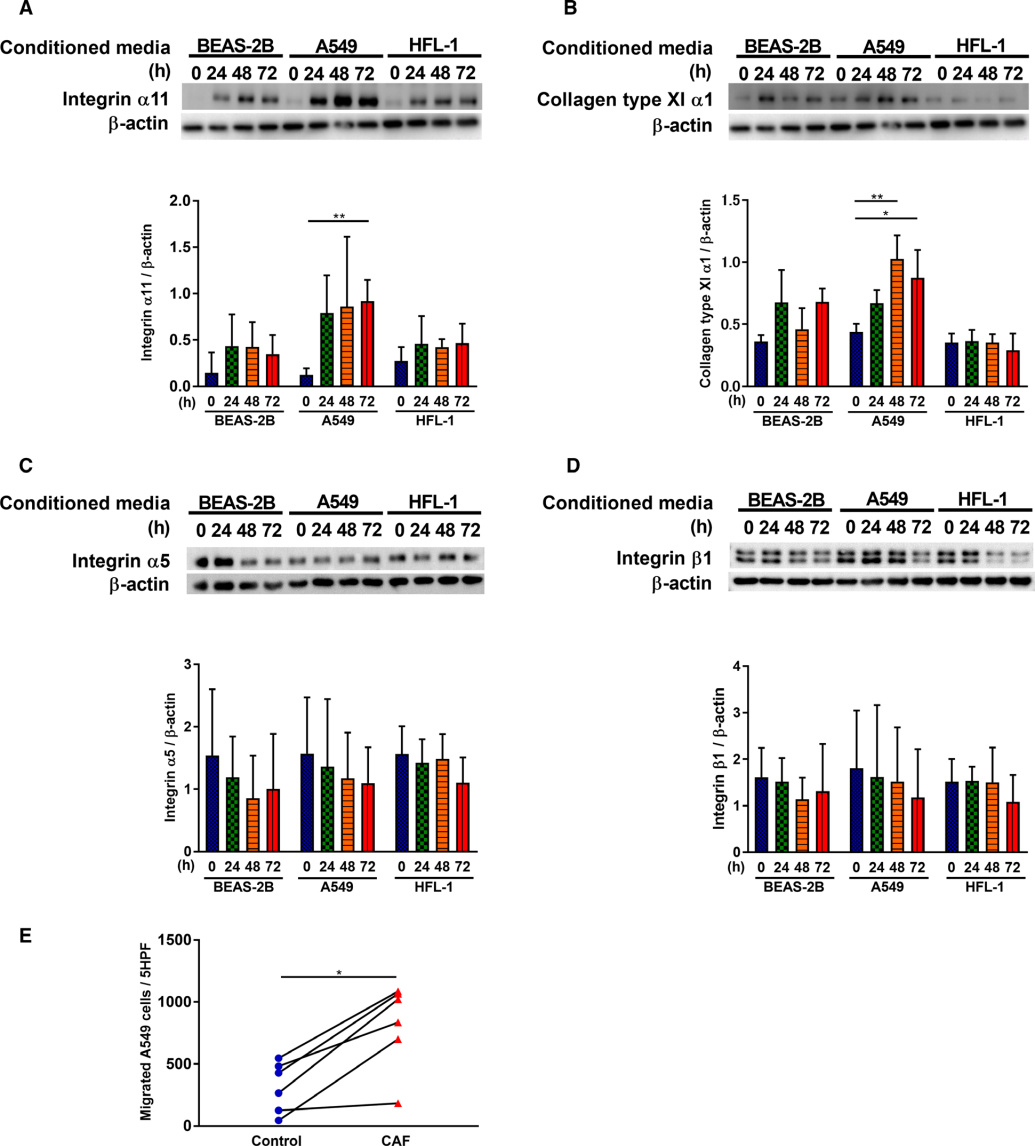
Phenotypic alteration of lung fibroblasts via fibroblast–lung cancer cell interaction. The effect of conditioned media on (A) ITGA11, (B) COL11A1, (C) integrin α5, and (D) integrin β1 expression levels in HFL‐1 cells. The conditioned media were harvested from BEAS‐2B as the normal epithelial cells, A549 as the lung adenocarcinoma cell line, and HFL‐1 as the negative control (see Section 2.5). (A‐D; *n* = 3, unpaired Student’s *t*‐test) Vertical axis: expression of protein normalized to β‐actin. (E) Sub‐confluent control fibroblasts and CAFs were cultured and the supernatant was harvested after being incubated for 48 h. Subsequently, the migration ability of A549 cells toward supernatant from CAFs was compared to that of the control fibroblasts. (E; *n* = 6, Wilcoxon) Vertical axis: number of migrated cells per 5HPF. Each symbol represents one patient. The values represent the mean ± SD. *P < 0.05, **P < 0.01.

Cancer‐associated fibroblasts are known to promote growth, invasion, and metastasis of cancer cells via CAF–cancer cell interactions [9]. Therefore, we assessed whether the phenotypic transformation of normal lung fibroblasts to CAFs occurred by examining the response of fibroblasts to conditioned media from A549 adenocarcinoma cells, as an *in vitro* fibroblast–cancer cell interaction model. Conditioned media from A549 cultures significantly stimulated ITGA11 expression in HFL‐1 cells (Fig. 7A: *P* = 0.007) at 72 h and COL11A1 expression at 48 and 72 h (Fig. 7B: 48 h; *P* = 0.007, 72 h; *P* = 0.0317) compared to treatment with media from BEAS‐2B cells, derived from normal bronchial epithelium, obtained from autopsy of noncancerous individuals, or HFL‐1 cells. However, conditioned media from A549 cultures did not stimulate integrin α5 or integrin β1 expression (Fig. 7C,D). Next, we assessed the migration of A549 cells induced by media harvested from control fibroblasts or CAFs. Harvested media from CAFs significantly stimulated migration (*P* = 0.0313) of A549 compared to that of the control fibroblasts (Fig. 7E).

### 3.8 Effect of *ITGA11* genetic modification on fibroblast features

**Fig. 8:**
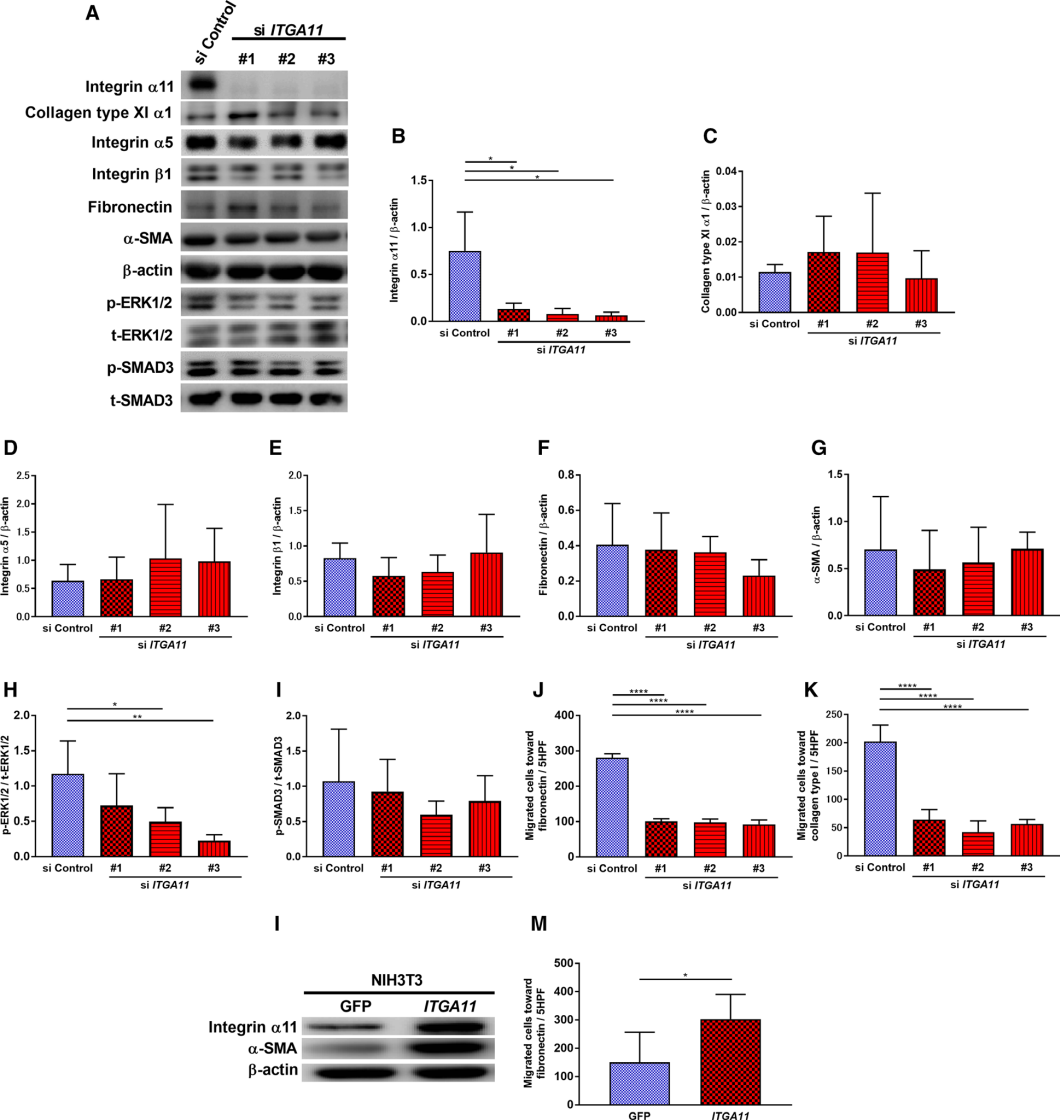
Effect of *ITGA11* genetic modification on fibroblast features. (A) *ITGA11* was knocked down using siRNA in HFL‐1 and proteins were extracted and subjected to western blot analysis to detect the expression levels of each target (see Section 2.6). Vertical axis: (B) ITGA11, (C) COL11A1, (D) integrin α5, (E) integrin β1, (F) fibronectin, and (G) α‐SMA protein expression normalized to β‐actin, (H) p‐ERK1/2 protein expression normalized to t‐ERK1/2 and (I) p‐SMAD3 protein expression normalized to t‐SMAD3. (J) Migration toward fibronectin (20 µg·mL^−1^) was assessed after silencing *ITGA11*. Vertical axis: number of migrated cells per 5HPF. (K) Migration toward collagen type I (1 µg·mL^−1^) was assessed after silencing *ITGA11*. (B‐K; *n* ≥ 3, one‐way ANOVA) Vertical axis: number of migrated cells per 5HPF. (L) NIH 3T3 cells were transfected with *ITGA11*‐overexpression vector or GFP and subjected to western blot analysis to detect the ITGA11 and α‐SMA expression levels (see Section 2.7). (M) Migration toward fibronectin (20 µg·mL^−1^) was assessed with *ITGA11*‐overexpressing NIH 3T3 cells. (M; *n* = 8, Mann–Whitney) Vertical axis: number of migrated cells per 5HPF. The values represent the mean ± SD. *P < 0.05, **P < 0.01, ****P < 0.0001.

To investigate the specific roles of ITGA11 in CAFs, we first knocked down *ITGA11* in HFL‐1. Silencing *ITGA11* (Fig. 8A,B) suppressed p‐ERK1/2 (Fig. 8H), migration toward fibronectin, and collagen type I (Fig. 8J,K). However, *ITGA11* silencing did not affect COL11A1 (Fig. 8C), integrin α5 (Fig. 8D) or integrin β1 (Fig. 8E), fibronectin (Fig. 8F), α‐SMA (Fig. 8G), or p‐SMAD3 (Fig. 8I) expression. To investigate the specific roles of ITGA11 in fibroblasts, we next overexpressed it in NIH 3T3 cells, which caused an increase in α‐SMA expression (Fig. 8L) and stimulated migration toward fibronectin (Fig. 8M).

### 3.9 Schematic illustration of acquired features of the phenotype of cancer‐associated fibroblasts

**Fig. 9:**
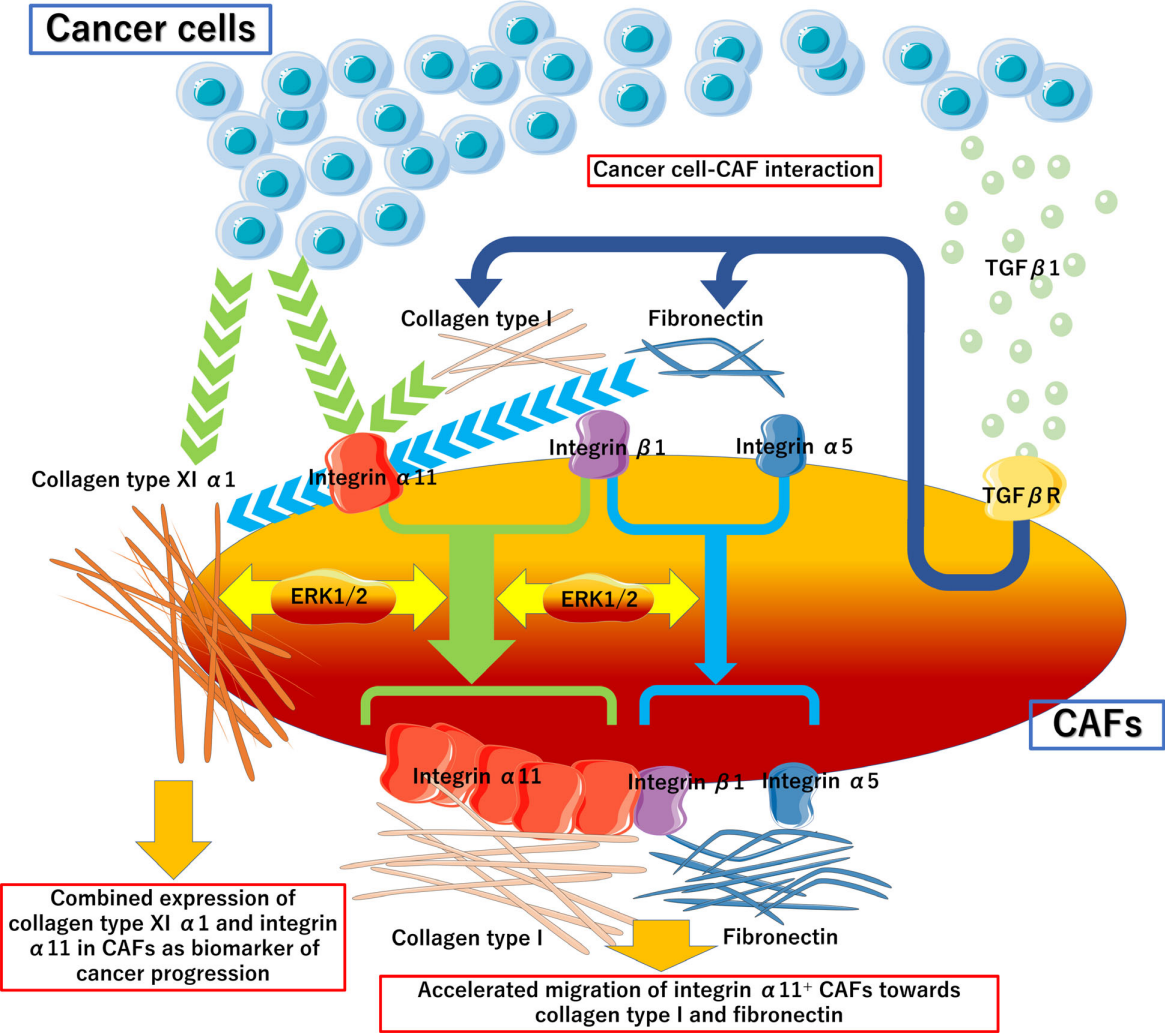
Schematic illustration of acquired features of the phenotype of cancer‐associated fibroblasts. TGF‐β1‐stimulated fibronectin and collagen type I production induce ITGA11 expression in fibroblasts, resulting in accelerated CAF‐mediated migration toward fibronectin and collagen type I via ERK1/2 signal without changing integrin α5 β1 expressions. The released fibronectin induced COL11A1 production, which was accompanied by ITGA11 expression via ERK1/2 signal in cancer stroma.

TGF‐β1‐stimulated fibronectin and collagen type I production induce ITGA11 expression in fibroblasts, resulting in accelerated CAF‐mediated migration toward fibronectin and collagen type I via ERK1/2 signal without changing integrin α5 β1 expressions. These recruitments of overexpressed ITGA11 CAFs into lung cancer stroma induce cancer cell migration through cancer cell–CAF interaction. The released fibronectin induced COL11A1 production, which was accompanied by ITGA11 expression via ERK1/2 signal in cancer stroma. This leads to further CAFs with specific phenotype: ‘ITGA11^+^/ COL11A1^+^ CAF’, through the CAF–cancer cell interaction (Fig. 9).

## 4 Discussion

In this study, we identified 390 enriched DEGs including the previously reported CAF marker, *ITGA11*, and several collagen subtypes including *COL11A1* and the representative ligand of ITGA11, *COL1A1*, as well as 121 depleted DEGs, in 16 paired samples of normal lung fibroblasts and CAFs derived from NSCLC patients. We also conducted a series of bioinformatics analyses on DEGs to explore CAF‐specific candidates associated with the increase in CAF migratory capacity. The GO analysis revealed that activated fibrotic processes, including ECM organization and cell adhesion, were enriched in CAFs, with specific DEGs including *ITGA11*, *COL1A1,* and *COL11A1* in ECM organization (Table S3). Hence, increase in the expression of ITGA11 may promote CAF migration toward fibronectin and collagen type I, although COL11A1 did not act as a direct chemoattractant for fibroblast‐mediated migration. Thus, CAFs are likely controlled by transcriptional regulatory networks distinct from those in normal lung fibroblasts.

In the present study, overexpressed ITGA11 in CAF, increased migration toward collagen type I, known as the representative ligand of ITGA11, was consistent in integrin receptor‐mediated migration. In the previous study, neutralizing anti‐integrin α5 and β1 antibodies for the blockade of fibronectin receptor partially attenuated TGF‐β1‐stimulated lung fibroblast migration toward fibronectin [51]. These results suggest that some other CAF‐specific functional mechanism, perhaps through ITGA11 regulation, may be involved in fibroblast migration. Interestingly, the increasing capacity of CAF‐mediated migration toward fibronectin was associated with upregulation of the ITGA11 via ERK1/2 signaling. Furthermore, pretreatment of CAFs with fibronectin or collagen type I, which induced ITGA11 in fibroblasts, significantly enhanced migration toward both collagen type I and fibronectin, compared to the control fibroblasts. These data suggest CAFs overexpressed ITGA11, causing a specific response in their migration toward fibronectin and collagen type I, whereas integrin α5 or β1 were not affected. Previous reports using CAFs demonstrated that specific integrin receptor subtypes, including integrin α5β1 and αVβ3, are receptors for organizing fibronectin to promote directional cancer cell invasion [52, 53]. Furthermore, integrin α5β1 and αVβ3 have been shown to cross talk with other integrins, such as integrin α6β1, and they synergistically enhance cell attachment [54, 55]. Therefore, although fibroblast migration toward fibronectin is thought to involve the fibronectin receptor, integrin α5β1, we have been unable to demonstrate the nature of the direct contribution. This study provides additional evidence that overexpression of ITGA11 in CAFs enhanced their migration toward fibronectin and collagen type I. Some differential integrin‐specific cross talk between integrin α5β1‐ and ITGA11‐mediated migration toward fibronectin may involve CAF‐specific functional bioactivity, and these mechanisms could be considered as unique CAF features.

The functions of ITGA11 related to fibrotic processes have previously been reported in *ITGA11* knockdown hepatic stellate cells, which resulted in reduced TGF‐β1‐induced differentiation and fibrotic characteristics, as indicated by loss of protrusions, attenuated adhesion and migration, and impaired contractility of collagen I matrices in these cells [45]. In addition, *ITGA11*‐deficient mice showed decreased α‐SMA expression co‐localized with ITGA11 in the cancer stroma. Stromal ITGA11 expression affects the tumorigenicity and metastatic potential of NSCLC cells, as ITGA11 overexpression in CAFs increases their interaction with cancer cells [17, 50, 56, 57]. We demonstrated that the genetic inhibition of *ITGA11* not only suppressed the lung fibroblast migration only toward collagen type I but also toward fibronectin without downregulation of neither fibronectin nor integrin subunits α5 and β1. Meanwhile, the genetic overexpression of ITGA11 stimulated α‐SMA expression and migration toward fibronectin. This result suggested that ITGA11^+^ CAFs were the representative crucial target among the heterogenetic activated myofibroblast features.

Our results are consistent with previous studies, which showed that TGF‐β1 induces *ITGA11* expression in lung fibroblasts [58], and when CAFs were exposed to TGF‐β1, we observed an increase in fibronectin release, and migration toward fibronectin in CAFs; however, the response of TGF‐β1 was not high as compared to control fibroblasts. The *ITGA11* promoter is involved in regulating the function of SMAD‐binding elements [24], while *ITGA11* expression in CAFs modulated transcriptional targets of the TGF‐β1 signaling pathway [25]. However, in this study, *ITGA11* siRNA suppressed ERK1/2 activation but did not regulate the canonical SMAD3 signal. Alternatively, tumor cell lysates from *ITGA11^−/−^* mice reduce the levels of activated ERK1/2 compared with those from *ITGA11^+/+^* mice [25]. Similarly, we demonstrated that ERK1/2 phosphorylation was increased in CAFs, while treatment with an ERK inhibitor, CAS 1049738‐54‐6, suppressed TGF‐β1‐induced lung fibroblast migration toward fibronectin and collagen type I, as ITGA11, COL11A1, and fibronectin expressions were reduced. Therefore, acceleration of CAF migration may be independent of the canonical TGF‐β1‐SMAD3 pathway, potentially via fibrotic lung fibroblast which did not show an enriched *ITGA11* in CAGE analysis, as we previously described [48]. TGF‐β1‐induced fibronectin also resulted in excessive deposition of COL11A1, accompanied by ITGA11 expression in the cancer stroma, resulting in a CAF‐specific phenotype: ITGA11^+^/ COL11A1^+^ CAFs.

Herein, we have validated that the cancer cell‐mediated education of fibroblasts results in a change of phenotype from normal fibroblasts to CAFs. Moreover, supernatant media from lung adenocarcinoma cells (A549) strongly induced ITGA11 and COL11A1 expression, compared to that from normal epithelial cells (BEAS‐2B), without affecting expression of integrin α5 or β1 in HFL‐1. Meanwhile, the supernatant from CAFs containing abundant fibronectin significantly stimulated cancer cells (A549) migration compared to that from control lung fibroblasts. Thus, we propose the CAF‐specific phenotype is activated through interactions between cancer cells and CAFs, which results in the development of tumors by accelerating the infiltration of both CAFs and cancer cells. Both ITGA11 and COL11A1 were highly expressed in cancer stroma compared to normal lung tissue and the expression level of ITGA11 correlated with that of COL11A1, suggesting interaction with the cancer stroma. Furthermore, overexpressed ITGA11 in cancer stroma was clinically associated with high recurrence following surgical resection, as well as with progression of pathological stage in NSCLC patients. Although whether CAFs are associated with good or poor prognosis remains unclear and is contradictory in different studies [59], these results provide strengthened evidence for cross‐regulation of ITGA11 and COL11A1 in cancer stroma, suggesting that ITGA11^+^/ COL11A1^+^ CAFs may be a new candidate biomarker for clinical outcomes based on CAF‐mediated tumorigenicity in patients with NSCLC.

## 5 Conclusions

To the best of our knowledge, this is the first study demonstrating altered gene expression in CAFs compared to normal fibroblasts using CAGE analysis. We showed that the altered phenotypes are likely caused by cancer cell–stromal fibroblast interactions. Our results provide additional evidence that increased fibronectin‐ and collagen type I‐induced ITGA11 expression promoted the CAF migration mechanism via ERK1/2 signaling with overexpression of COL11A1, the CAF‐mediated surrogate biomarker. In addition, overexpressed ITGA11 accompanied by COL11A1 expression in cancer stroma was associated with a poor clinical outcome. These may have contributed to further activation of the cancer stroma by the interaction of the infiltrating ITGA11^+^/ COL11A1^+^ CAFs with the cancer microenvironment. The ITGA11^+^/ COL11A1^+^ CAFs subtype, therefore, may serve as a key determinant for the progression of NSCLC and may be considered for novel anti‐tumor strategies through the blockade of cancer cell–stroma interactions.

## Conflict of interest

The authors declare no conflict of interest.

## Author contributions

MI (Iwai) and MT were responsible for cell culture, biochemical studies, and interpretation of results. ST designed the study, and ST, MI (Iwai), and MT wrote the manuscript. HK contributed to bioinformatics analyses and manuscript writing. YH, MI (Itoh), and HK prepared the CAGE results. ST, TO, MH, AO, YH, MI (Itoh), HK, KY, KS, and KT provided technical advice and assisted in the interpretation of results. ST, TO, KK, YN, JW, IS, KT, and SO recruited patients and obtained their written informed consent. KY kindly gifted the vector to overexpress the *ITGA11* gene and provided technical advice for overexpression. YK, AO, and YI prepared the vector to overexpress *ITGA11* in fibroblasts. All authors critically reviewed and contributed to the final manuscript.

### Peer Review

The peer review history for this article is available at https://publons.com/publon/10.1002/1878‐0261.12937.

## Supporting information


**Fig. S1.** Determination of integrin α11 and collagen type XI α1 expression.
**Fig. S2.** Immunohistochemical staining of collagen type XI α1 in NSCLC tissues and the clinical associations.
**Fig. S3.** Cancer‐associated fibroblasts‐mediated migration and collagen type XI α1 production.
**Fig. S4.** TGF‐β1 release from cancer‐associated fibroblasts.
**Fig. S5.** HFL‐1 migration associated with collagen type I and fibronectin.
**Table S1.** Clinical and demographic characteristics.
**Table S2** Cap analysis of gene expression (CAGE).
**Table S3.** Gene ontology analysis.

## Data Availability

All data related to this study are available from the corresponding author upon reasonable request.
