# Supplementary material for: Cancer‐associated fibroblast migration in non‐small cell lung cancers is modulated by increased integrin α11 expression
Source: Mol Oncol. 2021 Mar 25;15(5):1507–27. doi: 10.1002/1878-0261.12937 (PMC8096795; doi:10.1002/1878-0261.12937)
Supplement: Supplementary file 1 — Fig. S1. Determination of integrin α11 and collagen type XI α1 expression. Fig. S2. Immunohistochemical staining of collagen type XI α1 in NSCLC tissues and the clinical associations. Fig. S3. Cancer‐associated fibroblasts‐mediated migration and collagen type XI α1 production. Fig. S4. TGF‐β1 release from cancer‐associated fibroblasts. Fig. S5. HFL‐1 migration associated with collagen type I and fibronectin. Table S1. Clinical and demographic characteristics. Table S2 Cap analysis of gene expression (CAGE). Table S3. Gene ontology analysis. [file MOL2-15-1507-s001.docx]

**Overexpressed integrin α11 promotes cancer-associated fibroblast migration**

Moe Iwai, Miniwan Tulafu, Shinsaku Togo, Hideya Kawaji, [Kotaro Kadoya](https://www.ncbi.nlm.nih.gov/pubmed/?term=Kadoya%20K%5BAuthor%5D&cauthor=true&cauthor_uid=28284212), Yukiko Namba, Jin Jin, Junko Watanabe, Takahiro Okabe, Moulid Hidayat, Issei Sumiyoshi, Masayoshi Itoh, Yu Koyama, Yasuhiko Ito, Akira Orimo, Kazuya Takamochi, Shiaki Oh, Kenji Suzuki, Yoshihide Hayashizaki, Koji Yoshida, Kazuhisa Takahashi.

**Supporting Information**

**Supporting Figures**

**Fig. S1 Determination of integrin α11 and collagen type XI α1 expression**

**(a)** Immunohistochemical staining of human liver cancer tissue using anti-integrin α11 and staining of pancreatic ductal adenocarcinoma using anti-collagen type XI α1 as positive and IgG as negative controls (see Methods 2.4). Original magnification: 100×; Scale bars: 200 μm. **(b)** After sub-confluent HFL-1, A549, and fibroblasts from patient controls, as well as CAFs from affected patients, were cultured, proteins were extracted and analyzed by western blot to detect integrin α11 and collagen type XI α1.

**Fig. S2 Immunohistochemical staining of collagen type XI** α**1 in NSCLC tissues and the clinical associations**

**(a, b)** Association between recurrence and collagen type XI α1 expression in cancer stroma and cancer epithelium. (a, b; n = 25, Mann-Whitney) **(c, d)** Association between the pathological staging and collagen type XI α1 expression in cancer stroma and cancer epithelium. (c, d; n = 38, one-way ANOVA) Vertical axis: H-score values (see Methods 2.4). Each symbol represents one patient. The values represent the mean ± SD.

**Fig. S3 Cancer-associated fibroblasts-mediated migration and collagen type XI** α**1 production**

**(a)** Sub-confluent control fibroblasts and CAFs were cultured, the supernatant was harvested, and the collagen type XI α1 level was quantified. (a; n = 9, Mann-Whitney) Vertical axis: collagen type XI α1 production expressed as amount per mL. Each data point represents one patient. **(b)** Chemotaxis toward fibronectin was assessed using different concentrations of human collagen type XI α1. (b; n = 3, one-way ANOVA) Vertical axis: number of migrated cells per five high-power fields (5HPF). The values represent the mean ± SD. **P < 0.01.

**Fig. S4 TGF-β1 release from cancer-associated fibroblasts**

Sub-confluent control fibroblasts and CAFs were cultured and the supernatant was harvested. TGF-β1 production was quantified. (n = 18, Mann-Whitney) Vertical axis: TGF-β1 production expressed as amount per mL. Each data point represents one patient. The values represent the mean ± SD.

**Fig. S5 HFL-1 migration associated with collagen type I and fibronectin**

After HFL-1 were cultured and incubated with or without human collagen type I (1 µg mL^-1^) for 48 h or fibronectin (20 µg mL^-1^) for 8 h, migration toward human collagen type I (1 µg mL^-1^) or fibronectin (20 µg mL^-1^) was measured. (n = 3, unpaired Student’s *t*-test) Vertical axis: number of migrated cells per five high-power fields (5HPF). The values represent the mean ± SD. ****P < 0.0001.

**Supporting Tables**

**Table S1 Clinical and demographic characteristics**

| Parameter | No. of patients | Mean ± SD |
| --- | --- | --- |
| Age (years) | 16 | 63.9 ± 9.6 |
| Sex (male/female) | 13/3 |  |
| Smoking history (yes/no) | 12/4 |  |
| Stage (1/2/3/4) | 11/2/2/1 |  |
| Relapsed (yes/no) | 5/11 |  |
| Pathology (adenocarcinoma/squamous cell carcinoma) | 11/5 |  |
| Histological differentiation (high/middle/low) | 3/5/8 |  |

**Table S2** **Cap analysis of gene expression (CAGE)**

| CAF > Control (Log FC > 0) | LogFC | LogCPM | p-value | FDR |
| --- | --- | --- | --- | --- |
| chr14,107099659,107099670,-;p1@IGHV3-62 | 6.202814 | -0.26296 | 2.54E-05 | 0.003378 |
| chr16,65160001,65160033,-;p@chr16:65160001..65160033,- | 5.385329 | -0.02908 | 3E-07 | 0.000171 |
| chr9,100615499,100615519,+;p1@FOXE1 | 4.866196 | -0.24328 | 2.79E-05 | 0.003631 |
| chr16,65159972,65159987,-;p@chr16:65159972..65159987,- | 4.648534 | -1.16749 | 8.43E-06 | 0.001538 |
| chr11,2153071,2153082,-;p8@IGF2 | 4.517366 | -1.19926 | 4.36E-05 | 0.004836 |
| chr5,174252830,174252847,+;p@chr5:174252830..174252847,+ | 4.42646 | -0.10724 | 6.61E-15 | 1.77E-10 |
| chr12,33049691,33049756,-;p1@PKP2 | 4.410037 | -0.92291 | 3.47E-08 | 3.65E-05 |
| chr16,65159938,65159954,-;p@chr16:65159938..65159954,- | 4.370085 | -1.25149 | 4.11E-05 | 0.004663 |
| chr15,99433751,99433766,+;p14@IGF1R | 4.197227 | -0.84905 | 1.04E-05 | 0.001739 |
| chr1,77333155,77333184,+;p1@ST6GALNAC5 | 4.185005 | 2.429858 | 1.67E-10 | 7.12E-07 |
| chr1,173174706,173174733,-;p3@TNFSF4 | 3.984152 | -1.22571 | 6.19E-05 | 0.006195 |
| chr12,124779630,124779647,+;p1@FAM101A | 3.908645 | -1.0457 | 2.78E-05 | 0.003631 |
| chr21,44846975,44846992,-;p2@SIK1 | 3.898758 | -0.3784 | 7.72E-08 | 6.47E-05 |
| chr12,106532631,106532642,-;p9@NUAK1 | 3.889867 | -0.85708 | 5.3E-07 | 0.000247 |
| chr12,106532724,106532740,-;p1@NUAK1 | 3.849348 | 1.178671 | 5.03E-10 | 1.59E-06 |
| chr16,81737354,81737443,+;p@chr16:81737354..81737443,+ | 3.784864 | -0.56854 | 7.79E-06 | 0.00146 |
| **chr1,103574001,103574014,-;p3@COL11A1** | 3.779966 | 0.838952 | 9.64E-08 | 7.45E-05 |
| chr8,104384724,104384782,+;p3@CTHRC1 | 3.728396 | 0.276384 | 2.7E-07 | 0.000159 |
| chr1,12676766,12676799,-;p12@DHRS3 | 3.654501 | -0.77368 | 2.07E-06 | 0.000612 |
| chr13,38153485,38153498,-;p@chr13:38153485..38153498,- | 3.632584 | -0.07558 | 2.21E-07 | 0.000135 |
| chr11,2158507,2158526,-;p3@IGF2 | 3.546412 | 0.216928 | 7.82E-07 | 0.000309 |
| **chr15,68643106,68643126,-;p4@ITGA11** | 3.49869 | -1.2582 | 7.28E-05 | 0.006964 |
| chr8,70378852,70378866,+;p3@SULF1 | 3.49024 | -0.08978 | 4.06E-08 | 4.1E-05 |
| chr7,47622176,47622207,-;p2@TNS3 | 3.39159 | 1.924596 | 1.11E-08 | 1.63E-05 |
| chr4,78978468,78978516,+;p1@FRAS1 | 3.350992 | -0.15309 | 6.53E-09 | 1.22E-05 |
| chr12,71003881,71003904,-;p5@PTPRB | 3.340762 | -0.89457 | 2.22E-05 | 0.003014 |
| chr13,38154048,38154108,-;p@chr13:38154048..38154108,- | 3.333055 | -0.87398 | 3.88E-05 | 0.004468 |
| chr8,70378936,70378956,+;p5@SULF1 | 3.325885 | -0.11629 | 9.33E-07 | 0.000336 |
| chr20,44746917,44746933,+;p2@CD40 | 3.323174 | -0.55907 | 1.01E-07 | 7.45E-05 |
| chr8,70378989,70379039,+;p2@SULF1 | 3.311402 | 3.261917 | 3.38E-11 | 2.31E-07 |
| chr8,70379072,70379089,+;p4@SULF1 | 3.252675 | -0.13929 | 4.94E-07 | 0.000236 |
| chr12,106532657,106532694,-;p2@NUAK1 | 3.252534 | 0.5755 | 1.32E-07 | 9.18E-05 |
| chr1,203154383,203154466,-;p3@CHI3L1 | 3.231205 | -0.14097 | 1.52E-05 | 0.002304 |
| chr1,25256756,25256774,-;p1@RUNX3 | 3.230178 | -0.69753 | 1.26E-05 | 0.001986 |
| chr1,77333117,77333139,+;p2@ST6GALNAC5 | 3.187947 | 0.936847 | 2.46E-07 | 0.000146 |
| **chr1,103574024,103574063,-;p1@COL11A1** | 3.187631 | 2.089764 | 9.85E-08 | 7.45E-05 |
| chr17,43299241,43299290,+;p1@FMNL1 | 3.186737 | 0.251326 | 2.63E-10 | 9.83E-07 |
| chr4,183065793,183065864,+;p2@ODZ3 | 3.185005 | 1.298884 | 1.73E-10 | 7.12E-07 |
| chr6,116479897,116479942,-;p2@COL10A1 | 3.176647 | 0.647762 | 3.51E-07 | 0.000187 |
| chr5,152870255,152870270,+;p3@GRIA1 | 3.168177 | -0.6224 | 3.65E-06 | 0.000902 |
| chr5,147286093,147286107,-;p1@C5orf46 | 3.167811 | -0.39171 | 6.54E-06 | 0.001315 |
| chr15,68594466,68594499,-;p@chr15:68594466..68594499,- | 3.165413 | 0.191843 | 6.9E-06 | 0.001367 |
| chr1,13910194,13910339,+;p1@PDPN | 3.158521 | 2.891921 | 5.16E-06 | 0.001144 |
| chr4,183065548,183065568,-;p2@ENST00000315302 | 3.152207 | -0.63706 | 4.35E-06 | 0.000995 |
| chr22,45098067,45098143,+;p1@ARHGAP8,p1@PRR5-ARHGAP8,p1@PRR5 | 3.141417 | 0.306112 | 4.66E-10 | 1.59E-06 |
| chr11,77795673,77795689,-;p@chr11:77795673..77795689,- | 3.121546 | -0.67644 | 5.95E-06 | 0.001266 |
| chr8,22436956,22436968,+;p12@PDLIM2 | 3.120876 | -1.01679 | 6.3E-07 | 0.000264 |
| chr1,173445944,173445969,+;p4@PRDX6 | 3.109186 | -0.82136 | 1.68E-06 | 0.000514 |
| chr19,17799139,17799181,-;p1@UNC13A | 3.104985 | -0.80657 | 1.74E-05 | 0.00252 |
| chr13,38156549,38156595,-;p1@AK297914 | 3.070328 | -0.77448 | 3.29E-05 | 0.004009 |
| chr15,51669513,51669571,+;p2@GLDN | 3.05311 | -0.10244 | 1.06E-05 | 0.001758 |
| chr1,246023808,246023820,-;p@chr1:246023808..246023820,- | 3.052004 | 2.646548 | 8.72E-07 | 0.000331 |
| chr5,180230830,180230903,-;p2@MGAT1 | 3.041583 | -0.19401 | 8.48E-07 | 0.000328 |
| chr12,71003751,71003810,-;p2@PTPRB | 3.021114 | 0.022291 | 3.29E-07 | 0.000182 |
| chr8,22436635,22436650,+;p2@PDLIM2 | 3.020544 | 3.876359 | 1.32E-08 | 1.74E-05 |
| chr5,152870287,152870303,+;p4@GRIA1 | 3.017385 | -0.9016 | 4.08E-05 | 0.004655 |
| chr13,42265559,42265609,-;p@chr13:42265559..42265609,- | 3.017159 | -0.36706 | 0.000105 | 0.008857 |
| chr5,143584901,143584987,+;p1@KCTD16 | 3.016353 | -0.3086 | 4.3E-08 | 4.1E-05 |
| chr15,65715242,65715290,-;p1@IGDCC4 | 2.991543 | -0.47246 | 3.11E-05 | 0.003937 |
| chr12,54402729,54402743,+;p3@HOXC8 | 2.973728 | -0.83029 | 9.26E-06 | 0.001639 |
| **chr15,68724544,68724555,-;p3@ITGA11** | 2.964375 | 0.0894 | 1.11E-06 | 0.000377 |
| chr8,134203273,134203289,+;p1@WISP1 | 2.933804 | 2.342343 | 9.84E-08 | 7.45E-05 |
| chr18,56246785,56246806,-;p1@ALPK2 | 2.917804 | -0.72829 | 1.04E-05 | 0.001737 |
| chr2,85804663,85804748,+;p1@VAMP8 | 2.909738 | 3.199495 | 4.02E-12 | 4.12E-08 |
| chr13,38158135,38158197,-;p@chr13:38158135..38158197,- | 2.901625 | -0.484 | 6.35E-06 | 0.001288 |
| chr6,12957560,12957606,+;p6@PHACTR1 | 2.889061 | -0.62365 | 6.11E-06 | 0.001279 |
| chr15,68650846,68650865,-;p@chr15:68650846..68650865,- | 2.885082 | -0.83259 | 3.31E-05 | 0.00401 |
| **chr15,68724490,68724516,-;p1@ITGA11** | 2.882466 | 3.732153 | 8.37E-08 | 6.81E-05 |
| chr8,70539477,70539491,+;p@chr8:70539477..70539491,+ | 2.871402 | -0.59047 | 1.14E-05 | 0.001867 |
| chr9,101471138,101471256,-;p1@GABBR2 | 2.869403 | -1.08335 | 1.66E-05 | 0.002435 |
| chr1,9711781,9711813,+;p1@PIK3CD | 2.863136 | -0.51668 | 0.000102 | 0.008678 |
| chr1,55505184,55505247,+;p1@PCSK9 | 2.843507 | 0.236736 | 6.77E-05 | 0.006629 |
| chr16,3115626,3115648,+;p1@IL32 | 2.822063 | 1.539999 | 8.51E-05 | 0.007741 |
| chr5,152870215,152870251,+;p1@GRIA1 | 2.819011 | -0.53049 | 5.03E-06 | 0.001122 |
| chr9,137764479,137764484,-;p1@uc004cff.2 | 2.808747 | -0.93322 | 5.17E-05 | 0.005468 |
| chr5,5146295,5146366,+;p1@ADAMTS16 | 2.798602 | -0.8359 | 2.16E-05 | 0.002996 |
| chr8,70379043,70379054,+;p6@SULF1 | 2.786528 | -0.56237 | 4.06E-06 | 0.000956 |
| chr4,183370146,183370219,+;p1@ODZ3 | 2.782602 | 1.298378 | 1.03E-09 | 2.5E-06 |
| chr14,80678512,80678529,-;p1@DIO2 | 2.778259 | -0.16692 | 1.75E-07 | 0.000112 |
| chr3,154797843,154797858,+;p15@MME | 2.771054 | 0.16663 | 1.26E-06 | 0.000415 |
| chr4,77908899,77908914,+;p9@SEPT11 | 2.764111 | -0.76855 | 2.92E-06 | 0.000764 |
| **chr1,103574154,103574165,-;p4@COL11A1** | 2.763608 | 1.430543 | 4.49E-07 | 0.000219 |
| chr2,228028911,228028932,-;p1@COL4A4 | 2.750576 | -0.81998 | 9.8E-05 | 0.008411 |
| chr3,8811288,8811305,-;p1@OXTR | 2.738729 | 1.826054 | 7.79E-05 | 0.007248 |
| chr3,154798096,154798115,+;p3@MME | 2.727567 | 2.095419 | 6.07E-07 | 0.000257 |
| chr7,45953339,45953442,-;p@chr7:45953339..45953442,- | 2.715569 | 0.986813 | 1E-08 | 1.63E-05 |
| chr2,238343455,238343486,-;p1@ENST00000409910 | 2.712186 | -0.2851 | 1.42E-05 | 0.002195 |
| chr22,33255699,33255744,+;p4@TIMP3 | 2.697166 | 0.621012 | 2.73E-08 | 3.2E-05 |
| chr12,106532697,106532718,-;p4@NUAK1 | 2.690294 | 0.4312 | 9.34E-06 | 0.001643 |
| chr3,154798129,154798155,+;p5@MME | 2.682911 | 0.500389 | 2.53E-06 | 0.000704 |
| chr7,45953287,45953301,-;p@chr7:45953287..45953301,- | 2.678076 | 0.386877 | 5.95E-07 | 0.000257 |
| chr4,183065716,183065735,+;p7@ODZ3 | 2.667376 | -0.90549 | 9.07E-05 | 0.007994 |
| chr1,9299767,9299791,+;p9@H6PD | 2.667024 | -0.98272 | 4.62E-05 | 0.00503 |
| chr1,17307576,17307627,-;p4@MFAP2 | 2.655815 | -0.35831 | 1.29E-06 | 0.000419 |
| chr1,183247896,183247948,-;p3@NMNAT2 | 2.649209 | -0.7366 | 4.95E-05 | 0.005302 |
| chr12,71003840,71003875,-;p3@PTPRB | 2.643972 | -0.27877 | 1E-05 | 0.001702 |
| chr17,43298917,43298993,+;p3@FMNL1 | 2.64281 | -0.14419 | 4.86E-05 | 0.005233 |
| chr18,18822185,18822213,+;p1@GREB1L | 2.642489 | -0.63243 | 7.19E-06 | 0.001393 |
| chr13,38158993,38159017,-;p@chr13:38158993..38159017,- | 2.637903 | -0.52012 | 5.47E-05 | 0.005694 |
| chr7,47622210,47622231,-;p9@TNS3 | 2.63641 | 0.358398 | 4.35E-07 | 0.000218 |
| chr19,38755462,38755531,+;p3@SPINT2 | 2.633864 | 1.74022 | 6.65E-13 | 9.1E-09 |
| chr11,77794459,77794475,+;p@chr11:77794459..77794475,+ | 2.632789 | 0.822449 | 9.37E-06 | 0.001643 |
| **chr15,68724518,68724531,-;p2@ITGA11** | 2.63226 | 2.009561 | 4.26E-08 | 4.1E-05 |
| chr19,46105411,46105481,-;p1@GPR4,p2@OPA3 | 2.610014 | 0.181238 | 1.7E-06 | 0.000516 |
| **chr1,103574068,103574099,-;p2@COL11A1** | 2.601579 | 1.808936 | 3.39E-07 | 0.000184 |
| chr15,33023745,33023757,+;p11@GREM1 | 2.592936 | 0.166404 | 9.31E-10 | 2.39E-06 |
| chr1,173176462,173176477,-;p1@TNFSF4 | 2.589938 | 0.428176 | 6.53E-05 | 0.006437 |
| chr7,98467629,98467645,-;p2@TMEM130 | 2.579758 | -0.63327 | 3.44E-06 | 0.000866 |
| chr3,154797326,154797389,+;p8@MME | 2.577689 | 0.501202 | 8.88E-09 | 1.52E-05 |
| chr8,134203303,134203314,+;p2@WISP1 | 2.575197 | 0.050187 | 3.97E-06 | 0.000941 |
| chr4,183065109,183065122,+;p5@ODZ3 | 2.567741 | -0.72537 | 4.2E-05 | 0.004696 |
| chr10,122216707,122216790,+;p1@PPAPDC1A | 2.564485 | 1.640425 | 2.69E-09 | 5.8E-06 |
| chr1,220863696,220863752,+;p1@C1orf115 | 2.552605 | 0.910431 | 1.49E-06 | 0.000467 |
| chr17,48261541,48261567,-;p@chr17:48261541..48261567,- | 2.548007 | -0.47596 | 0.000112 | 0.00926 |
| chr1,203155823,203155838,-;p1@CHI3L1 | 2.547187 | 7.586616 | 0.0001 | 0.008563 |
| chr10,48601574,48601591,-;p@chr10:48601574..48601591,- | 2.540901 | 0.364379 | 5.76E-07 | 0.000257 |
| chr4,183065584,183065617,-;p1@ENST00000315302 | 2.523194 | -0.56596 | 9.5E-06 | 0.001646 |
| chr2,85806236,85806245,+;p3@VAMP8 | 2.515298 | 0.284758 | 2.31E-06 | 0.000661 |
| chr1,246023875,246023930,-;p@chr1:246023875..246023930,- | 2.514333 | 3.159049 | 4.57E-06 | 0.001036 |
| chr18,29078131,29078218,+;p1@DSG2 | 2.512573 | 1.44785 | 7.13E-06 | 0.001393 |
| chr7,150037272,150037283,-;p@chr7:150037272..150037283,- | 2.51182 | 0.48297 | 2.14E-06 | 0.000617 |
| chr13,38153005,38153062,-;p@chr13:38153005..38153062,- | 2.505179 | -0.60887 | 7.49E-05 | 0.007115 |
| chr13,38172896,38172911,-;p1@POSTN | 2.503448 | 7.01101 | 1.49E-07 | 9.84E-05 |
| chr22,27620539,27620570,-;p2@ENST00000418271 | 2.50201 | -0.04296 | 1.09E-06 | 0.000376 |
| chr7,45952458,45952470,-;p12@IGFBP3 | 2.499267 | -0.19356 | 7.05E-06 | 0.001384 |
| chr2,162931082,162931100,-;p9@DPP4 | 2.487504 | -0.6947 | 9.62E-05 | 0.008327 |
| chr8,23159605,23159623,-;p@chr8:23159605..23159623,- | 2.487009 | -1.05239 | 1.88E-05 | 0.002703 |
| chr7,45953003,45953022,-;p17@IGFBP3 | 2.476683 | -1.12707 | 9.52E-05 | 0.008273 |
| chr19,38755237,38755321,+;p2@SPINT2 | 2.461691 | 2.389482 | 1.06E-10 | 5.45E-07 |
| chr8,70488248,70488262,+;p8@SULF1 | 2.460414 | 1.12959 | 2.27E-05 | 0.003061 |
| chr12,131438443,131438461,+;p1@GPR133 | 2.460104 | 0.755287 | 3.4E-05 | 0.004071 |
| chr1,245986269,245986299,-;p@chr1:245986269..245986299,- | 2.443653 | 0.087526 | 1.51E-06 | 0.000469 |
| chr5,174151612,174151633,+;p2@MSX2 | 2.437568 | -0.34547 | 6.25E-06 | 0.001285 |
| chr12,5542952,5542976,-;p@chr12:5542952..5542976,- | 2.426498 | -1.17629 | 6.54E-05 | 0.006437 |
| chr5,174151553,174151610,+;p1@MSX2 | 2.41335 | 0.206353 | 1.97E-08 | 2.37E-05 |
| chr7,2727817,2727881,+;p1@AMZ1 | 2.412849 | 0.007899 | 1.48E-06 | 0.000466 |
| chr15,33023180,33023193,+;p15@GREM1 | 2.405604 | -0.14427 | 7.72E-07 | 0.000307 |
| chr3,112358632,112358646,-;p5@CCDC80 | 2.396314 | 0.562717 | 5.71E-06 | 0.001233 |
| chr7,45952665,45952679,-;p8@IGFBP3 | 2.394824 | -0.63146 | 3.21E-05 | 0.003989 |
| chr15,74466870,74466923,+;p1@ISLR | 2.389684 | 1.097083 | 1.04E-05 | 0.001737 |
| chr15,33023199,33023239,+;p7@GREM1 | 2.381039 | 0.0806 | 5.72E-08 | 5E-05 |
| chr6,159590423,159590438,+;p1@FNDC1 | 2.375073 | 0.00529 | 8.34E-05 | 0.007625 |
| **chr17,48272818,48272860,-;p49@COL1A1** | 2.372188 | -0.96709 | 2.2E-05 | 0.003003 |
| chr2,109271481,109271493,+;p6@LIMS1 | 2.370938 | 1.265922 | 5.68E-11 | 3.33E-07 |
| chr10,28966443,28966461,+;p1@BAMBI | 2.367716 | 2.531565 | 3.75E-06 | 0.000916 |
| chr11,77791110,77791123,-;p3@NDUFC2,p4@NDUFC2-KCTD14 | 2.365458 | 1.52929 | 1.41E-08 | 1.81E-05 |
| chr17,79533608,79533634,-;p2@NPLOC4 | 2.36284 | -0.12076 | 4.14E-05 | 0.004671 |
| chr1,107683775,107683816,+;p2@NTNG1 | 2.361966 | 0.093707 | 6.52E-08 | 5.57E-05 |
| chr1,39624513,39624523,+;p@chr1:39624513..39624523,+ | 2.360779 | -1.13244 | 0.000101 | 0.008618 |
| chr6,134498969,134499006,-;p2@SGK1 | 2.356239 | -0.79632 | 6.37E-06 | 0.001288 |
| chr1,160990954,160990973,-;p3@F11R | 2.348397 | -0.62886 | 2.88E-06 | 0.000764 |
| chr3,112051994,112052043,+;p1@CD200 | 2.347594 | 0.739615 | 8.92E-05 | 0.007919 |
| chr7,45956182,45956271,-;p5@IGFBP3 | 2.346839 | -0.37138 | 5.56E-05 | 0.005739 |
| chr22,33255512,33255535,+;p5@TIMP3 | 2.33878 | 2.189852 | 3.11E-06 | 0.000798 |
| chr15,93198942,93199035,-;p1@FAM174B | 2.334587 | -0.03107 | 0.000124 | 0.009966 |
| chr3,154797877,154797889,+;p12@MME | 2.333059 | 0.290345 | 2.93E-05 | 0.003761 |
| chr22,33255360,33255398,+;p2@TIMP3 | 2.3218 | 2.049863 | 9.29E-07 | 0.000336 |
| chr22,33256388,33256401,+;p@chr22:33256388..33256401,+ | 2.321738 | 1.115997 | 4.82E-06 | 0.001081 |
| chr7,15725757,15725828,-;p5@MEOX2 | 2.320328 | -0.78842 | 3.36E-05 | 0.00406 |
| chr1,156863470,156863521,+;p1@PEAR1 | 2.320002 | 3.423836 | 7.04E-12 | 5.77E-08 |
| chr4,78978751,78978764,+;p2@FRAS1 | 2.314569 | -0.67306 | 2.09E-05 | 0.002922 |
| chr11,77790833,77790844,-;p2@NDUFC2,p3@NDUFC2-KCTD14 | 2.310009 | 1.839826 | 8.64E-15 | 1.77E-10 |
| chr22,33257962,33257975,+;p19@TIMP3 | 2.307911 | 0.567114 | 5.24E-06 | 0.001156 |
| chr11,77791156,77791159,-;p4@NDUFC2,p5@NDUFC2-KCTD14 | 2.28445 | -0.58364 | 6.25E-06 | 0.001285 |
| chr11,75479781,75479808,+;p2@DGAT2 | 2.276413 | 0.139512 | 1.33E-07 | 9.18E-05 |
| chr18,7117741,7117752,-;p3@LAMA1 | 2.229232 | -0.54306 | 3.28E-05 | 0.004009 |
| chr13,32605546,32605589,+;p6@FRY | 2.202485 | -0.27276 | 2.78E-06 | 0.000745 |
| chr13,78492955,78492973,-;p1@EDNRB | 2.201224 | 0.389094 | 8.79E-05 | 0.007876 |
| chr1,215256585,215256622,+;p6@KCNK2 | 2.199766 | -0.0301 | 7.59E-05 | 0.007117 |
| chr2,28201109,28201125,+;p2@BRE | 2.185031 | 0.396901 | 1.22E-08 | 1.72E-05 |
| chr3,154797454,154797484,+;p2@MME | 2.18318 | 2.38061 | 3.1E-08 | 3.44E-05 |
| chrX,12156829,12156879,+;p1@FRMPD4 | 2.17893 | -0.50338 | 3.14E-05 | 0.003948 |
| chr12,106532595,106532615,-;p5@NUAK1 | 2.170624 | -0.24707 | 2.04E-05 | 0.002867 |
| chr7,15726243,15726293,-;p2@MEOX2 | 2.16514 | -0.37814 | 2.79E-05 | 0.003631 |
| chr1,17307629,17307653,-;p5@MFAP2 | 2.157338 | -0.46557 | 7.51E-06 | 0.001439 |
| chr4,159093538,159093547,-;p10@FAM198B | 2.154728 | -0.71576 | 5.22E-05 | 0.005501 |
| chr7,45954422,45954464,-;p4@IGFBP3 | 2.153892 | -0.18402 | 7.15E-05 | 0.006867 |
| chr7,45960863,45960877,-;p1@IGFBP3 | 2.151187 | 7.165137 | 2.43E-07 | 0.000146 |
| chr4,88896857,88896874,+;p1@SPP1 | 2.141787 | 0.147053 | 8.13E-06 | 0.001496 |
| chr2,162930622,162930641,-;p4@DPP4 | 2.140774 | 3.541759 | 2.52E-06 | 0.000704 |
| chr7,94060122,94060134,+;p@chr7:94060122..94060134,+ | 2.138537 | -0.10937 | 4.16E-06 | 0.00097 |
| chr8,70405073,70405088,+;p1@SULF1 | 2.121673 | 5.784171 | 1.44E-05 | 0.002215 |
| chr22,27620603,27620700,-;p1@ENST00000418271,p1@ENST00000444114 | 2.118697 | 1.130001 | 6.89E-07 | 0.000283 |
| chr14,79745715,79745740,+;p2@NRXN3 | 2.114298 | 1.413154 | 2.58E-06 | 0.000704 |
| chr17,74379267,74379297,+;p4@SPHK1 | 2.110382 | -0.38345 | 5.9E-05 | 0.005945 |
| chr3,122044084,122044106,+;p1@CSTA | 2.106429 | 2.734925 | 0.000111 | 0.009235 |
| chr13,32605483,32605496,+;p8@FRY | 2.106184 | -0.54058 | 3.16E-05 | 0.003966 |
| chr22,33257682,33257717,+;p12@TIMP3 | 2.095099 | 1.154004 | 0.000105 | 0.00887 |
| chr9,137591918,137591938,+;p@chr9:137591918..137591938,+ | 2.093708 | -0.62532 | 2.22E-05 | 0.003014 |
| chr22,33255285,33255302,+;p9@TIMP3 | 2.09305 | -0.29109 | 0.000101 | 0.008595 |
| chr10,28966416,28966437,+;p2@BAMBI | 2.09257 | 1.017117 | 9.17E-07 | 0.000336 |
| chr3,116164306,116164380,-;p1@LSAMP | 2.091487 | 1.662762 | 3.05E-07 | 0.000172 |
| chr3,154797700,154797725,+;p4@MME | 2.088002 | 2.124083 | 4.61E-07 | 0.000223 |
| chr9,101017634,101017649,-;p4@TBC1D2 | 2.075586 | 2.719605 | 1.02E-07 | 7.45E-05 |
| chr10,26727647,26727722,+;p2@APBB1IP | 2.066528 | 0.056394 | 5.49E-05 | 0.005708 |
| chr16,3115386,3115412,+;p2@IL32 | 2.052658 | 1.429989 | 4.21E-05 | 0.004696 |
| chr2,162930861,162930874,-;p10@DPP4 | 2.039249 | 0.415228 | 3.8E-05 | 0.004406 |
| chr7,15726296,15726315,-;p1@MEOX2 | 2.036248 | 0.311518 | 8.52E-06 | 0.001548 |
| chr3,154797636,154797696,+;p1@MME | 2.035933 | 3.031974 | 7.38E-09 | 1.32E-05 |
| chr22,33254044,33254070,+;p@chr22:33254044..33254070,+ | 2.035501 | 1.907587 | 7.56E-06 | 0.001444 |
| chr5,33892081,33892101,-;p1@ADAMTS12 | 2.033937 | 2.862019 | 5.05E-08 | 4.6E-05 |
| chr15,33024989,33024999,+;p17@GREM1 | 2.033644 | 1.637994 | 2.59E-06 | 0.000704 |
| chr7,47834880,47834900,+;p2@C7orf69 | 2.032541 | -0.28532 | 0.000114 | 0.009394 |
| chr16,3115324,3115364,+;p3@IL32 | 2.026072 | 2.529905 | 1.88E-06 | 0.000566 |
| chr3,154797781,154797800,+;p6@MME | 2.009829 | 3.096247 | 3.7E-06 | 0.00091 |
| chr18,5543988,5544019,-;p1@EPB41L3 | 2.004715 | 3.098061 | 1.06E-08 | 1.63E-05 |
| chr2,202098203,202098256,+;p3@CASP8 | 2.001652 | -0.69805 | 1.15E-05 | 0.001878 |
| chr19,38755042,38755091,+;p5@SPINT2 | 1.987923 | 0.254663 | 7.47E-07 | 0.000303 |
| **chr17,48262913,48262927,-;p9@COL1A1** | 1.979891 | 1.512716 | 1.23E-06 | 0.000407 |
| chr19,782761,782833,+;p@chr19:782761..782833,+ | 1.976663 | -0.54488 | 5.03E-05 | 0.005373 |
| chr21,43639211,43639279,+;p1@ABCG1 | 1.976103 | 0.976015 | 8.89E-06 | 0.001587 |
| chr22,33254028,33254041,+;p@chr22:33254028..33254041,+ | 1.967627 | 0.94334 | 2.82E-05 | 0.003667 |
| **chr17,48263174,48263189,-;p2@COL1A1** | 1.959576 | 4.793171 | 3.29E-05 | 0.004009 |
| chr1,117452491,117452579,+;p1@PTGFRN | 1.955611 | 0.615102 | 5.7E-06 | 0.001233 |
| chr1,193155729,193155757,-;p1@B3GALT2 | 1.954123 | 0.188887 | 9.44E-05 | 0.008226 |
| chr1,156863447,156863464,+;p2@PEAR1 | 1.945373 | -0.13089 | 1E-05 | 0.001702 |
| chr3,154797486,154797518,+;p10@MME | 1.94373 | -0.53887 | 4.69E-05 | 0.005074 |
| chr2,162929965,162930004,-;p11@DPP4 | 1.942361 | -0.0231 | 0.000111 | 0.009235 |
| chr4,95679072,95679125,+;p1@BMPR1B | 1.941791 | 0.20168 | 3.03E-09 | 6.04E-06 |
| chr4,77908859,77908883,+;p5@SEPT11 | 1.929822 | -0.22587 | 4.68E-05 | 0.005074 |
| chr16,19896150,19896205,-;p1@GPRC5B | 1.919643 | 1.873041 | 5.59E-07 | 0.000252 |
| chr22,33255620,33255635,+;p10@TIMP3 | 1.913527 | 1.472365 | 6.06E-05 | 0.006084 |
| chr1,215256074,215256113,+;p1@KCNK2 | 1.91165 | 1.557135 | 1.63E-06 | 0.000503 |
| chr20,54987220,54987233,+;p6@CASS4 | 1.909074 | -0.47403 | 2.87E-05 | 0.003712 |
| chr7,47621844,47621855,-;p7@TNS3 | 1.904586 | 1.257499 | 3.09E-09 | 6.04E-06 |
| chr17,36608636,36608650,-;p2@ENST00000429284 | 1.902901 | 0.49438 | 2.03E-05 | 0.002867 |
| chr12,53343633,53343696,-;p2@KRT8 | 1.902121 | 1.090238 | 3.91E-06 | 0.000938 |
| chr7,45952925,45952936,-;p14@IGFBP3 | 1.900819 | -0.69422 | 7E-05 | 0.006794 |
| chr18,7117813,7117843,-;p2@LAMA1 | 1.900039 | 1.758578 | 4.13E-07 | 0.000212 |
| chr17,48262517,48262540,-;p@chr17:48262517..48262540,- | 1.89987 | 2.976717 | 1E-05 | 0.001702 |
| chr7,29234375,29234464,+;p1@CHN2 | 1.899048 | 0.074546 | 6.63E-05 | 0.006509 |
| chr16,3115299,3115312,+;p5@IL32 | 1.89181 | 0.585991 | 3.75E-05 | 0.004382 |
| chr19,47287990,47288086,-;p2@SLC1A5 | 1.890719 | 1.42985 | 5.58E-07 | 0.000252 |
| chr8,22442505,22442531,+;p@chr8:22442505..22442531,+ | 1.887972 | -0.43994 | 2.36E-05 | 0.003172 |
| chr3,128208039,128208096,+;p1@ENST00000464242,p1@ENST00000468377,p1@uc003ekp.2 | 1.887187 | 0.325492 | 9.44E-06 | 0.001646 |
| chr15,33025032,33025060,+;p8@GREM1 | 1.877476 | 1.694444 | 5.17E-05 | 0.005468 |
| chr4,7941440,7941455,-;p2@AFAP1 | 1.862571 | 0.947188 | 7.75E-06 | 0.001458 |
| chr7,151433342,151433366,-;p5@PRKAG2 | 1.853101 | 0.488044 | 3.39E-05 | 0.004071 |
| chr12,52301280,52301299,+;p2@ACVRL1 | 1.843712 | 0.326137 | 4.08E-05 | 0.004655 |
| chr15,33025787,33025805,+;p@chr15:33025787..33025805,+ | 1.842623 | 0.961364 | 0.00012 | 0.009713 |
| chr3,25469598,25469601,-;p@chr3:25469598..25469601,- | 1.84045 | -0.3398 | 4.06E-05 | 0.004652 |
| chr9,101017871,101017897,-;p2@TBC1D2 | 1.836005 | 2.735689 | 3.2E-05 | 0.003989 |
| chr2,202098175,202098202,+;p2@CASP8 | 1.834539 | -0.12338 | 8.89E-06 | 0.001587 |
| chr15,33023286,33023299,+;p4@GREM1 | 1.819899 | 1.487986 | 4.46E-07 | 0.000219 |
| chr15,33023250,33023262,+;p19@GREM1 | 1.817099 | -0.36899 | 8.59E-05 | 0.007798 |
| chr15,33023323,33023333,+;p20@GREM1 | 1.816575 | -0.47573 | 1.24E-05 | 0.001976 |
| chr15,33022885,33022907,+;p12@GREM1 | 1.814839 | 0.222876 | 5.4E-05 | 0.005668 |
| chr21,44846999,44847017,-;p1@SIK1 | 1.812962 | 2.434976 | 6.4E-05 | 0.006361 |
| chr2,189876466,189876506,+;p1@AF130082 | 1.798399 | 2.97255 | 0.000114 | 0.009394 |
| chr18,7117785,7117811,-;p1@LAMA1 | 1.791091 | 2.489653 | 7.89E-05 | 0.00726 |
| chr18,5543961,5543982,-;p5@EPB41L3 | 1.782861 | -0.05068 | 1.2E-05 | 0.001937 |
| chr18,61554932,61554949,+;p1@SERPINB2 | 1.780429 | 5.457031 | 0.000114 | 0.009394 |
| chr6,2903534,2903552,-;p1@SERPINB9 | 1.777196 | 2.881633 | 1.86E-08 | 2.32E-05 |
| chr15,33025271,33025279,+;p@chr15:33025271..33025279,+ | 1.7617 | 1.750217 | 5.17E-05 | 0.005468 |
| chr4,30723003,30723054,+;p3@PCDH7 | 1.752953 | 0.126491 | 1.27E-05 | 0.001996 |
| chr5,159343481,159343528,+;p1@ADRA1B | 1.740954 | 0.137565 | 5.52E-05 | 0.005719 |
| chr15,33026325,33026338,+;p@chr15:33026325..33026338,+ | 1.736207 | 1.547913 | 0.000119 | 0.009713 |
| chr17,48262548,48262561,-;p@chr17:48262548..48262561,- | 1.725795 | 4.720131 | 1.99E-05 | 0.002841 |
| chr17,48261877,48261964,-;p@chr17:48261877..48261964,- | 1.725686 | 6.358499 | 1.18E-05 | 0.001912 |
| chr19,18717589,18717616,-;p1@CRLF1 | 1.721115 | 1.969813 | 8.63E-06 | 0.001561 |
| chr19,10397621,10397642,+;p1@ICAM4 | 1.720814 | 0.388826 | 3.82E-05 | 0.004418 |
| chr22,33255587,33255606,+;p6@TIMP3 | 1.71277 | 2.015699 | 0.000116 | 0.009519 |
| chr8,72756637,72756664,-;p3@MSC | 1.710788 | 0.729919 | 1.01E-05 | 0.001708 |
| chr17,54671047,54671075,+;p1@NOG | 1.710324 | 0.804734 | 3.66E-05 | 0.00431 |
| chr20,54987494,54987512,+;p2@CASS4 | 1.706373 | 3.318863 | 6.45E-07 | 0.000267 |
| chr3,154797428,154797450,+;p9@MME | 1.691611 | 1.20589 | 7.58E-07 | 0.000305 |
| **chr17,48277133,48277317,-;p4@COL1A1** | 1.68318 | 4.359826 | 9.01E-07 | 0.000336 |
| chr13,32605767,32605790,+;p2@FRY | 1.67849 | 0.867536 | 1.42E-06 | 0.000453 |
| chr7,75401513,75401535,-;p1@CCL26 | 1.677723 | 1.488808 | 3.93E-06 | 0.000939 |
| chr18,5544022,5544045,-;p4@EPB41L3 | 1.664678 | 1.216532 | 3.16E-08 | 3.44E-05 |
| chr18,44790480,44790556,+;p@chr18:44790480..44790556,+ | 1.663401 | 1.87423 | 1.41E-05 | 0.002193 |
| chr2,220174072,220174149,-;p1@PTPRN | 1.660751 | 3.094361 | 5.72E-05 | 0.005862 |
| **chr17,48266783,48266900,-;p13@COL1A1** | 1.65874 | 2.211561 | 5.74E-05 | 0.005862 |
| chr15,94774773,94774830,+;p1@MCTP2 | 1.658342 | 0.942472 | 1.11E-06 | 0.000377 |
| chr15,33024106,33024122,+;p9@GREM1 | 1.652857 | 1.797755 | 4.06E-05 | 0.004652 |
| chrX,31285042,31285103,-;p1@DMD | 1.651313 | 0.697391 | 4.11E-06 | 0.000963 |
| chr17,48265264,48265325,-;p1@Y15912,p1@Y15914 | 1.65014 | 3.030874 | 1.24E-05 | 0.001976 |
| chr3,141105795,141105814,+;p@chr3:141105795..141105814,+ | 1.649499 | 0.780016 | 5.98E-07 | 0.000257 |
| chr17,48261749,48261844,-;p@chr17:48261749..48261844,- | 1.635956 | 2.956589 | 9.33E-05 | 0.008164 |
| chr3,112357575,112357598,-;p@chr3:112357575..112357598,- | 1.628057 | 1.045888 | 1.7E-05 | 0.002474 |
| chr8,23261625,23261641,-;p1@LOXL2 | 1.623024 | 8.224934 | 1.64E-07 | 0.000107 |
| chr7,94059589,94059687,+;p1@K02046 | 1.611593 | 3.075148 | 2.91E-06 | 0.000764 |
| chr15,33023095,33023177,+;p2@GREM1 | 1.610095 | 4.094444 | 3.31E-05 | 0.00401 |
| chr19,34287174,34287222,+;p@chr19:34287174..34287222,+ | 1.606682 | 0.987701 | 7.12E-05 | 0.006867 |
| **chr17,48264102,48264133,-;p8@COL1A1** | 1.603227 | 1.386875 | 9.44E-05 | 0.008226 |
| chr11,62456995,62457013,-;p1@LRRN4CL | 1.601271 | 4.258598 | 4.42E-05 | 0.004871 |
| chr4,30721263,30721290,+;p@chr4:30721263..30721290,+ | 1.591294 | 0.357998 | 1.32E-05 | 0.002059 |
| chr5,151047094,151047114,-;p9@SPARC | 1.574473 | 1.225504 | 9.08E-05 | 0.007994 |
| chr8,57472137,57472150,-;p1@ENST00000519144 | 1.573645 | -0.13513 | 8.84E-05 | 0.007902 |
| chr3,112359634,112359653,-;p10@CCDC80 | 1.570171 | 1.893716 | 5.78E-06 | 0.001241 |
| chr15,33010194,33010211,+;p1@GREM1 | 1.562798 | 6.654114 | 5.82E-05 | 0.005916 |
| chr5,33892102,33892117,-;p3@ADAMTS12 | 1.55916 | 1.01749 | 9.72E-05 | 0.008358 |
| chr5,151042725,151042748,-;p@chr5:151042725..151042748,- | 1.556848 | 0.649601 | 1.21E-05 | 0.001939 |
| chr15,80843484,80843499,+;p5@ARNT2 | 1.554383 | 0.018529 | 8.63E-05 | 0.00782 |
| chr8,54625130,54625159,+;p2@ENST00000517688 | 1.552517 | 3.467757 | 2.48E-05 | 0.00331 |
| chr12,88813035,88813063,-;p@chr12:88813035..88813063,- | 1.548911 | 1.194355 | 8.89E-07 | 0.000335 |
| chr5,53999131,53999158,-;p@chr5:53999131..53999158,- | 1.536672 | 0.785286 | 4.11E-05 | 0.004663 |
| chrX,133683665,133683681,-;p@chrX:133683665..133683681,- | 1.531889 | 2.278384 | 5.75E-05 | 0.005862 |
| **chr17,48272098,48272147,-;p19@COL1A1** | 1.515164 | 0.991357 | 0.000122 | 0.009864 |
| chr22,33197683,33197698,+;p1@TIMP3 | 1.506512 | 8.440723 | 1.36E-06 | 0.000439 |
| chr15,33023069,33023091,+;p5@GREM1 | 1.497736 | 3.75441 | 1.45E-05 | 0.002225 |
| chr17,36608652,36608681,-;p1@ENST00000429284 | 1.493876 | 1.896729 | 4.34E-06 | 0.000995 |
| **chr17,48262750,48262761,-;p23@COL1A1** | 1.493838 | 3.189886 | 7.17E-05 | 0.00687 |
| chr16,70835017,70835028,-;p3@VAC14 | 1.480774 | 0.667496 | 0.000107 | 0.008925 |
| chr17,74497432,74497516,-;p1@RHBDF2 | 1.48004 | 1.912461 | 3.58E-06 | 0.000895 |
| chr20,54987258,54987289,+;p3@CASS4 | 1.47147 | 0.437682 | 5.87E-06 | 0.001255 |
| chr5,121414004,121414025,-;p3@LOX | 1.471229 | 3.192238 | 4.37E-06 | 0.000995 |
| chr2,162930180,162930226,+;p@chr2:162930180..162930226,+ | 1.464475 | 0.209416 | 8.28E-05 | 0.007587 |
| chr5,151046007,151046075,-;p1@AK126525 | 1.453144 | 1.17686 | 1.48E-05 | 0.002254 |
| chr12,5541267,5541285,+;p1@NTF3 | 1.441214 | 2.222239 | 7.96E-05 | 0.007309 |
| chr8,104383728,104383756,+;p2@CTHRC1 | 1.440656 | 4.47164 | 4.1E-08 | 4.1E-05 |
| chr20,54987305,54987337,+;p1@CASS4 | 1.434177 | 2.064032 | 3.68E-05 | 0.004318 |
| chr4,30722217,30722226,+;p4@PCDH7 | 1.419721 | 0.37399 | 6.85E-05 | 0.006686 |
| chr15,63354769,63354809,+;p8@TPM1 | 1.415021 | 1.666365 | 2.18E-05 | 0.002996 |
| chr5,121413171,121413182,-;p5@LOX | 1.401816 | 2.232198 | 6.27E-06 | 0.001285 |
| chr8,72756267,72756296,-;p4@MSC | 1.396374 | 2.243921 | 6.86E-05 | 0.006686 |
| chr18,33767473,33767498,+;p1@MOCOS | 1.393938 | 2.158895 | 6.73E-06 | 0.001346 |
| chr5,151043733,151043775,-;p@chr5:151043733..151043775,- | 1.381276 | 1.22386 | 8.79E-05 | 0.007876 |
| chr17,74380683,74380733,+;p1@SPHK1 | 1.374446 | 3.435368 | 1.6E-05 | 0.002396 |
| chr3,145879113,145879126,-;p2@PLOD2 | 1.371106 | 3.179957 | 2.18E-05 | 0.002996 |
| chr8,104383700,104383709,+;p4@CTHRC1 | 1.36301 | 1.331746 | 7.67E-06 | 0.001451 |
| chr8,72756667,72756736,-;p2@MSC | 1.362021 | 1.470406 | 5.47E-06 | 0.001193 |
| chr9,38392661,38392678,+;p2@ALDH1B1 | 1.36184 | 0.63891 | 0.000117 | 0.009568 |
| chr2,201994552,201994568,+;p12@CFLAR | 1.349971 | -0.37366 | 0.000124 | 0.009966 |
| chr11,66083196,66083252,-;p2@CD248 | 1.347279 | 1.36467 | 1.03E-06 | 0.000363 |
| chr5,121413702,121413713,-;p7@LOX | 1.340353 | 1.628208 | 2.11E-06 | 0.000617 |
| chr5,121413974,121413986,-;p4@LOX | 1.337015 | 4.049249 | 1.05E-06 | 0.000365 |
| chr7,47577036,47577094,-;p@chr7:47577036..47577094,- | 1.332305 | 0.718288 | 3.67E-05 | 0.00431 |
| chr3,112359244,112359263,-;p11@CCDC80 | 1.325764 | 1.67035 | 9.53E-06 | 0.001646 |
| chr15,63334938,63334957,+;p1@TPM1 | 1.32575 | 8.568542 | 1.38E-07 | 9.25E-05 |
| chr11,128563948,128564003,+;p1@FLI1 | 1.323669 | 1.60687 | 0.0001 | 0.008563 |
| chr15,63414051,63414069,+;p2@LACTB | 1.323243 | 3.019112 | 9.11E-06 | 0.001618 |
| chr9,137533678,137533693,+;p2@COL5A1 | 1.322713 | 3.571678 | 7.65E-06 | 0.001451 |
| chr3,112357162,112357195,-;p6@CCDC80 | 1.314812 | 3.279389 | 0.000106 | 0.008885 |
| chr8,104383759,104383821,+;p1@CTHRC1 | 1.306732 | 6.901169 | 2.96E-06 | 0.00077 |
| chr15,31283767,31283793,-;p3@MTMR10 | 1.299549 | 0.433269 | 9.03E-05 | 0.007989 |
| chr9,104249319,104249398,-;p2@C9orf125 | 1.289714 | 2.522598 | 3.6E-06 | 0.000895 |
| chr2,46524897,46524911,+;p2@EPAS1 | 1.275213 | 0.657587 | 6.47E-05 | 0.006401 |
| chr3,112359880,112359894,-;p2@CCDC80 | 1.27059 | 5.868651 | 1.61E-05 | 0.0024 |
| chr18,71959140,71959154,-;p2@CYB5A | 1.269495 | 1.140753 | 1.95E-06 | 0.000581 |
| chr8,56792377,56792404,+;p1@LYN | 1.262357 | 2.348883 | 1.14E-05 | 0.001867 |
| chr19,47291843,47291859,-;p1@SLC1A5 | 1.261454 | 7.193048 | 2.14E-05 | 0.002972 |
| **chr17,48278983,48278999,-;p1@COL1A1** | 1.261042 | 12.69988 | 1.55E-05 | 0.002332 |
| chr6,139694819,139694929,-;p3@CITED2 | 1.238551 | 1.526936 | 1.09E-08 | 1.63E-05 |
| chr5,121413944,121413965,-;p1@LOX | 1.211696 | 6.183892 | 8.55E-07 | 0.000328 |
| chr8,72756213,72756243,-;p5@MSC | 1.211358 | 1.520801 | 2.9E-05 | 0.003744 |
| chr10,50323510,50323541,-;p2@VSTM4 | 1.199153 | 1.017184 | 3.29E-05 | 0.004009 |
| chr8,72756063,72756125,-;p1@MSC | 1.198807 | 1.199364 | 9.66E-05 | 0.008327 |
| chr19,38810447,38810529,+;p1@KCNK6 | 1.182954 | 3.092568 | 2.56E-06 | 0.000704 |
| chr7,143079112,143079140,+;p18@ZYX | 1.178954 | 1.323742 | 7.52E-05 | 0.007115 |
| chr17,62207487,62207511,-;p1@ERN1 | 1.165262 | 2.052082 | 8.21E-06 | 0.001504 |
| chr2,69240547,69240561,+;p4@ANTXR1 | 1.164417 | 3.859525 | 0.000113 | 0.009315 |
| chr19,38746979,38747050,-;p1@PPP1R14A | 1.161422 | 6.177264 | 9.89E-05 | 0.008474 |
| chr13,73633131,73633149,+;p1@KLF5 | 1.159904 | 2.362968 | 7.81E-05 | 0.007248 |
| chr17,46627545,46627560,+;p@chr17:46627545..46627560,+ | 1.14823 | 1.880683 | 8.47E-05 | 0.007724 |
| chr5,121414045,121414063,-;p2@LOX | 1.144631 | 3.891671 | 2.03E-05 | 0.002867 |
| chr5,151066514,151066529,-;p3@SPARC | 1.134723 | 7.465762 | 3.16E-06 | 0.000805 |
| chr9,110045636,110045647,+;p4@RAD23B | 1.120878 | 2.146929 | 1.27E-05 | 0.001994 |
| chr1,86043992,86044008,-;p7@DDAH1 | 1.096036 | 2.139917 | 1.64E-05 | 0.002423 |
| chr5,151043787,151043805,-;p@chr5:151043787..151043805,- | 1.092274 | 3.124099 | 7.5E-05 | 0.007115 |
| chr18,33877654,33877706,+;p1@FHOD3 | 1.091306 | 1.226421 | 3.78E-05 | 0.004394 |
| chr8,120651070,120651091,-;p1@ENPP2 | 1.08983 | 3.498075 | 3.25E-05 | 0.004009 |
| chr5,179780312,179780343,-;p1@GFPT2 | 1.08534 | 4.967865 | 3.12E-05 | 0.003939 |
| chr10,105428481,105428540,-;p5@SH3PXD2A | 1.07973 | 2.351515 | 3.81E-06 | 0.000926 |
| chr5,121412855,121412875,-;p8@LOX | 1.068873 | 1.846983 | 6.93E-05 | 0.006738 |
| chr2,218808771,218808802,-;p1@TNS1 | 1.059674 | 4.597907 | 3.17E-05 | 0.003966 |
| chr19,676385,676403,+;p1@FSTL3 | 1.053385 | 4.839928 | 4.38E-05 | 0.004843 |
| chr1,151512775,151512792,+;p1@TUFT1 | 1.053326 | 2.835675 | 5.76E-05 | 0.005862 |
| chr1,86043864,86043895,-;p3@DDAH1 | 1.0512 | 3.717489 | 3.03E-05 | 0.003867 |
| chr5,33892118,33892144,-;p2@ADAMTS12 | 1.029542 | 1.871419 | 5.1E-05 | 0.005435 |
| chr5,95158375,95158485,-;p1@GLRX | 1.012463 | 7.319563 | 8.47E-08 | 6.81E-05 |
| chr3,112359959,112359972,-;p3@CCDC80 | 1.012295 | 4.909421 | 1.99E-05 | 0.002841 |
| chr5,95297513,95297527,-;p3@ELL2 | 0.998 | 4.664849 | 1.62E-05 | 0.002412 |
| chr8,42396839,42396860,-;p1@SLC20A2 | 0.995295 | 4.773949 | 3.59E-05 | 0.004244 |
| chr11,86666427,86666450,-;p1@FZD4 | 0.987461 | 3.671695 | 4.61E-05 | 0.005026 |
| chr1,86043758,86043791,-;p5@DDAH1 | 0.959879 | 1.438613 | 9.22E-05 | 0.008088 |
| chr11,121971132,121971166,-;p1@MIR100HG | 0.955862 | 6.495138 | 0.000104 | 0.008816 |
| chr6,43737939,43737956,+;p1@VEGFA | 0.954027 | 5.51649 | 4.33E-05 | 0.00481 |
| chr7,27135773,27135822,+;p1@HOTAIRM1 | 0.951995 | 4.0244 | 1E-06 | 0.000355 |
| chr1,86043808,86043860,-;p2@DDAH1 | 0.942494 | 4.406886 | 8.88E-05 | 0.007902 |
| chr5,95297464,95297507,-;p2@ELL2 | 0.881458 | 2.486676 | 9.55E-05 | 0.008282 |
| chr4,7941518,7941593,-;p1@AFAP1 | 0.877484 | 5.23038 | 2.17E-05 | 0.002996 |
| chr4,41216492,41216550,-;p1@APBB2 | 0.84149 | 4.019572 | 4.24E-07 | 0.000215 |
| chr7,151329215,151329358,-;p1@PRKAG2 | 0.827638 | 2.919984 | 2.77E-05 | 0.003631 |
| chr15,63414017,63414049,+;p1@LACTB | 0.801513 | 4.021863 | 3.41E-05 | 0.004071 |
| chr6,139695775,139695795,-;p1@CITED2 | 0.773229 | 8.377877 | 0.000103 | 0.008741 |
| chr5,125759064,125759131,+;p1@GRAMD3 | 0.754199 | 3.772131 | 1.47E-05 | 0.00224 |
| chr11,46299539,46299620,+;p2@CREB3L1 | 0.729829 | 3.470299 | 3.25E-05 | 0.004009 |
| chr8,131455835,131455928,-;p1@ASAP1 | 0.673009 | 4.507427 | 4.14E-05 | 0.004671 |
| chr19,16187296,16187344,+;p1@TPM4 | 0.659809 | 8.962243 | 7.58E-05 | 0.007117 |
| **CAF < Control (Log FC < 0)** | **LogFC** | **LogCPM** | **p-value** | **FDR** |
| chr18,22932080,22932170,-;p1@ZNF521 | -0.68988 | 3.050519 | 6.27E-05 | 0.006241 |
| chr1,148928291,148928331,+;p1@ENST00000294715,p1@ENST00000420597,p1@ENST00000452399 | -0.76976 | 5.122228 | 7.61E-05 | 0.007117 |
| chr15,67357924,67357952,+;p2@SMAD3 | -0.81993 | 5.196055 | 2.25E-05 | 0.003041 |
| chrX,38663529,38663546,+;p1@MID1IP1 | -0.875 | 4.685817 | 2.94E-05 | 0.003761 |
| chr14,95982953,95982999,-;p@chr14:95982953..95982999,- | -0.88356 | 3.126485 | 4.56E-05 | 0.004992 |
| chr10,102820989,102821005,+;p2@KAZALD1 | -0.98085 | 1.363235 | 3.4E-05 | 0.004071 |
| chr8,106330696,106330719,+;p2@ZFPM2 | -0.99025 | 1.979692 | 8.69E-05 | 0.00784 |
| chr10,81892415,81892447,+;p2@PLAC9 | -1.01413 | 5.155045 | 2.57E-05 | 0.003407 |
| chr3,143567262,143567343,-;p1@SLC9A9 | -1.02649 | 2.997774 | 2.42E-05 | 0.003251 |
| chr16,86601443,86601455,-;p@chr16:86601443..86601455,- | -1.03119 | 1.775808 | 4.83E-05 | 0.005215 |
| chr12,6309517,6309575,+;p1@CD9 | -1.04339 | 8.302733 | 8.48E-07 | 0.000328 |
| chr17,75372307,75372357,+;p25@SEPT9 | -1.04466 | 2.970179 | 3.28E-05 | 0.004009 |
| chr21,27011711,27011766,+;p2@JAM2 | -1.06419 | 1.456887 | 0.000117 | 0.009527 |
| chrX,107683009,107683088,+;p1@COL4A5 | -1.08875 | 3.025947 | 1.05E-05 | 0.00175 |
| chr17,59477197,59477212,+;p2@TBX2 | -1.1064 | 4.604131 | 7.03E-05 | 0.006802 |
| chr16,56666549,56666559,+;p5@MT1M | -1.11807 | 2.410528 | 5.42E-05 | 0.005672 |
| chr3,10206567,10206582,+;p2@IRAK2 | -1.12043 | 0.247955 | 9.69E-06 | 0.001663 |
| chr11,115375126,115375140,-;p1@CADM1 | -1.12083 | 5.751448 | 7.4E-05 | 0.007061 |
| chr12,115121770,115121781,-;p4@TBX3 | -1.12293 | 3.709161 | 6.24E-05 | 0.006229 |
| chr12,76425368,76425384,-;p1@PHLDA1 | -1.13145 | 8.472318 | 1.34E-07 | 9.18E-05 |
| chr1,171810606,171810649,+;p1@DNM3 | -1.18581 | 0.661504 | 0.000124 | 0.009997 |
| chr7,117513540,117513587,-;p2@CTTNBP2 | -1.1909 | 1.061483 | 7.75E-05 | 0.007231 |
| chr5,124084493,124084524,-;p2@ZNF608 | -1.20281 | 0.508783 | 1.88E-05 | 0.002703 |
| chr3,69134080,69134101,+;p2@ARL6IP5 | -1.20449 | 2.194744 | 2.79E-05 | 0.003631 |
| chr5,124082322,124082364,-;p1@ZNF608 | -1.22545 | 1.254757 | 6.04E-06 | 0.001277 |
| chr12,93966441,93966473,+;p3@SOCS2 | -1.24127 | 1.320496 | 0.00012 | 0.009713 |
| chr16,56716373,56716390,+;p1@MT1X | -1.2465 | 9.235034 | 5.43E-06 | 0.001191 |
| chr17,19314532,19314563,+;p1@RNF112 | -1.2546 | 1.854702 | 3.05E-05 | 0.003881 |
| chr7,117513474,117513500,-;p1@CTTNBP2 | -1.26713 | 1.170139 | 5.87E-05 | 0.005935 |
| chr11,70963809,70963836,-;p2@SHANK2 | -1.27199 | 1.594508 | 7.14E-05 | 0.006867 |
| chr16,57662527,57662556,+;p1@GPR56 | -1.29513 | 2.099051 | 0.00011 | 0.009208 |
| chr9,123165478,123165498,-;p2@CDK5RAP2 | -1.29948 | 2.051606 | 7.89E-05 | 0.00726 |
| chr17,33390667,33390762,-;p2@RFFL | -1.31037 | 0.159012 | 8.74E-05 | 0.007863 |
| chr1,6418966,6419024,-;p8@ACOT7 | -1.34206 | 2.731906 | 9.55E-06 | 0.001646 |
| chr10,30025857,30025872,-;p2@SVIL | -1.34376 | 3.294584 | 0.000121 | 0.009762 |
| chr6,154831779,154831801,-;p1@CNKSR3 | -1.35195 | 1.927692 | 5.57E-08 | 4.97E-05 |
| chr13,114540888,114540910,+;p@chr13:114540888..114540910,+ | -1.36996 | 1.394818 | 2.92E-06 | 0.000764 |
| chr10,81892478,81892493,+;p1@PLAC9 | -1.37354 | 4.41561 | 4.35E-06 | 0.000995 |
| chr10,81923408,81923450,-;p@chr10:81923408..81923450,- | -1.37684 | 2.764511 | 1.67E-05 | 0.002435 |
| chr5,142780237,142780261,-;p7@NR3C1 | -1.41257 | -0.1369 | 9.65E-05 | 0.008327 |
| chr3,12200368,12200494,-;p1@TIMP4 | -1.42369 | 2.157794 | 3.52E-05 | 0.00418 |
| chr16,86612174,86612188,+;p4@FOXL1 | -1.43879 | 2.178917 | 1.14E-05 | 0.001867 |
| chr16,57662566,57662582,+;p6@GPR56 | -1.45019 | 0.904035 | 5.57E-05 | 0.005739 |
| chr12,24715538,24715575,-;p1@ENST00000540811 | -1.45246 | 0.313045 | 0.000116 | 0.009515 |
| chr2,101767836,101767881,-;p1@TBC1D8 | -1.45693 | 0.142625 | 3.46E-05 | 0.004117 |
| chr13,114542074,114542079,-;p@chr13:114542074..114542079,- | -1.50808 | -0.39535 | 8.86E-05 | 0.007902 |
| chr8,106330543,106330554,+;p4@ZFPM2 | -1.50879 | 0.580544 | 9.23E-07 | 0.000336 |
| chr5,124082279,124082319,-;p3@ZNF608 | -1.52095 | -0.15185 | 5.57E-07 | 0.000252 |
| chr17,17399701,17399716,-;p1@RASD1 | -1.53222 | 0.621127 | 7.51E-05 | 0.007115 |
| chr5,124080203,124080258,-;p5@ZNF608 | -1.56897 | 0.030712 | 1.18E-05 | 0.001912 |
| chr3,187009468,187009531,-;p1@MASP1 | -1.58513 | 4.952275 | 5.46E-05 | 0.005694 |
| chr5,110409170,110409189,+;p6@TSLP | -1.59187 | -0.27832 | 2.05E-05 | 0.002869 |
| chr8,106330515,106330525,+;p6@ZFPM2 | -1.64297 | -0.41697 | 6.13E-06 | 0.001279 |
| chr11,62380709,62380748,+;p2@ROM1 | -1.64321 | 0.011852 | 2.57E-06 | 0.000704 |
| chr16,56666563,56666580,+;p2@MT1M | -1.66992 | 5.891343 | 1.26E-05 | 0.001986 |
| chr6,37665651,37665666,-;p1@MDGA1 | -1.69034 | 2.569016 | 1.97E-07 | 0.000125 |
| chr8,95273168,95273192,-;p2@GEM | -1.69966 | 0.941832 | 7.46E-06 | 0.001437 |
| chr16,5115913,5115977,-;p1@C16orf89 | -1.71914 | 1.481988 | 1.64E-05 | 0.002423 |
| chr8,27491402,27491476,+;p1@SCARA3 | -1.73233 | 6.997122 | 2.13E-07 | 0.000133 |
| chr12,115121802,115121814,-;p5@TBX3 | -1.75756 | 3.28689 | 6.45E-10 | 1.88E-06 |
| chr3,193853927,193853944,+;p1@HES1 | -1.81044 | 0.859685 | 5.87E-05 | 0.005935 |
| chr11,130320015,130320031,-;p1@AK123049 | -1.82854 | -0.18041 | 7.86E-06 | 0.001466 |
| chr1,145525015,145525048,+;p1@ITGA10 | -1.93714 | 0.248473 | 3.05E-06 | 0.000787 |
| chr20,9496574,9496598,+;p@chr20:9496574..9496598,+ | -1.94394 | -0.56581 | 7.83E-05 | 0.007252 |
| chr4,81118647,81118666,+;p1@PRDM8 | -1.94796 | 4.025974 | 4.84E-08 | 4.51E-05 |
| chr3,133748762,133748778,-;p1@SLCO2A1 | -1.95694 | 1.781174 | 4.48E-05 | 0.004925 |
| chr8,27472209,27472225,-;p1@CLU | -1.9972 | 11.90221 | 4.73E-06 | 0.001067 |
| chr10,123804015,123804053,+;p@chr10:123804015..123804053,+ | -2.01146 | -0.25036 | 3.76E-05 | 0.004387 |
| chr8,32504622,32504647,+;p7@NRG1 | -2.01519 | -0.25341 | 2.94E-05 | 0.003761 |
| chr11,846180,846233,+;p@chr11:846180..846233,+ | -2.02208 | -0.19848 | 1.21E-06 | 0.000405 |
| chr1,3528034,3528077,-;p2@MEGF6 | -2.05244 | -0.21932 | 9.66E-07 | 0.000345 |
| chr11,130318529,130318547,+;p1@ADAMTS15 | -2.05715 | 4.127743 | 3.19E-08 | 3.44E-05 |
| chr12,15103541,15103620,-;p6@ARHGDIB | -2.06255 | -0.12694 | 0.000108 | 0.009043 |
| chr9,102584128,102584144,+;p3@NR4A3 | -2.07858 | 0.875229 | 3.87E-06 | 0.000935 |
| chr13,86373448,86373468,-;p3@SLITRK6 | -2.11097 | -0.11922 | 7.57E-05 | 0.007117 |
| chr8,67039329,67039377,+;p1@TRIM55 | -2.12817 | 2.962284 | 7.93E-06 | 0.001472 |
| chr8,27469196,27469294,-;p2@CLU | -2.14461 | 1.078535 | 9.19E-05 | 0.008079 |
| chr1,11751574,11751662,-;p3@MAD2L2 | -2.17956 | 0.542951 | 4.16E-05 | 0.004674 |
| chr8,27463932,27463992,-;p4@CLU | -2.18802 | 1.320836 | 1.91E-05 | 0.00274 |
| chr16,27413488,27413517,+;p1@IL21R | -2.19505 | -0.32866 | 6.08E-07 | 0.000257 |
| chr16,27413815,27413889,+;p4@IL21R | -2.20988 | -0.07905 | 2.32E-06 | 0.000661 |
| chr2,11052080,11052117,+;p1@KCNF1 | -2.24324 | 1.088682 | 1.21E-06 | 0.000405 |
| chr8,27455711,27455732,-;p@chr8:27455711..27455732,- | -2.29862 | -0.08151 | 6.14E-06 | 0.001279 |
| chr20,9495392,9495403,+;p9@LAMP5 | -2.33485 | 0.208416 | 2.04E-05 | 0.002867 |
| chr13,86373510,86373529,-;p1@SLITRK6 | -2.33785 | 0.707759 | 1.67E-05 | 0.002435 |
| chr8,27463995,27464033,-;p15@CLU | -2.37352 | 0.577611 | 2.44E-06 | 0.000691 |
| chr2,241938025,241938046,+;p1@SNED1 | -2.38009 | 2.661371 | 6.93E-06 | 0.001367 |
| chr11,68452002,68452019,+;p1@GAL | -2.38777 | 6.008034 | 1.42E-06 | 0.000453 |
| chrX,85403445,85403479,+;p2@DACH2 | -2.45389 | 0.691752 | 4.56E-05 | 0.004992 |
| chr7,83278414,83278504,-;p1@SEMA3E | -2.45965 | -0.56716 | 8.13E-06 | 0.001496 |
| chr20,9495321,9495340,+;p8@LAMP5 | -2.46153 | 0.770953 | 2.13E-06 | 0.000617 |
| chrX,138287176,138287192,-;p1@FGF13 | -2.46703 | 0.301486 | 2.88E-07 | 0.000166 |
| chr20,9495410,9495426,+;p5@LAMP5 | -2.47297 | 1.326567 | 6.78E-06 | 0.00135 |
| chr8,27491533,27491544,+;p3@SCARA3 | -2.47383 | 0.845426 | 1.26E-08 | 1.72E-05 |
| chr20,9495263,9495313,+;p3@LAMP5 | -2.52913 | 2.701831 | 4.95E-05 | 0.005302 |
| chr5,44389148,44389161,-;p8@FGF10 | -2.53973 | -0.20287 | 8.69E-05 | 0.00784 |
| chr20,9495209,9495256,+;p1@LAMP5 | -2.54199 | 2.528121 | 2.68E-05 | 0.003553 |
| chr8,27468052,27468067,-;p13@CLU | -2.58627 | 3.868968 | 1.89E-06 | 0.000566 |
| chr20,9495194,9495205,+;p7@LAMP5 | -2.6392 | -0.21715 | 1.55E-05 | 0.002332 |
| chr2,183731355,183731384,-;p3@FRZB | -2.64718 | 3.086211 | 6.43E-05 | 0.006372 |
| chr5,44389509,44389536,-;p1@FGF10 | -2.65437 | 2.512633 | 4.18E-05 | 0.004686 |
| chr13,86373476,86373501,-;p4@SLITRK6 | -2.68068 | 0.561652 | 6.01E-07 | 0.000257 |
| chr3,69171739,69171754,-;p1@LMOD3 | -2.69365 | -0.8582 | 7.61E-05 | 0.007117 |
| chr8,27468075,27468130,-;p7@CLU | -2.72551 | 1.823718 | 5.04E-07 | 0.000238 |
| chr5,170846640,170846660,+;p1@FGF18 | -2.87886 | 2.468173 | 6.86E-10 | 1.88E-06 |
| chr6,69345166,69345259,+;p1@BAI3 | -2.8865 | -0.92441 | 5.74E-05 | 0.005862 |
| chr22,44482539,44482567,+;p14@PARVB | -3.12115 | -1.28025 | 9.01E-05 | 0.007989 |
| chr8,27468262,27468277,-;p27@CLU | -3.38243 | -0.78307 | 3.36E-06 | 0.000852 |
| chr8,27466559,27466566,+;p@chr8:27466559..27466566,+ | -3.41038 | -0.8774 | 7.85E-05 | 0.007253 |
| chr20,9495486,9495495,+;p13@LAMP5 | -3.42147 | 0.01241 | 3.41E-07 | 0.000184 |
| chrX,107683096,107683121,+;p3@COL4A5 | -3.43081 | 0.31989 | 3.86E-07 | 0.0002 |
| chr8,67039452,67039469,+;p2@TRIM55 | -3.48871 | -0.77133 | 1.42E-05 | 0.002195 |
| chrX,144899416,144899486,+;p3@SLITRK2 | -3.4888 | -1.1134 | 2.77E-06 | 0.000745 |
| chr16,56701968,56701983,-;p1@MT1G | -3.49897 | 5.838023 | 1.37E-09 | 3.12E-06 |
| chrX,107681633,107681692,-;p1@COL4A6 | -3.60072 | 0.953514 | 1.33E-07 | 9.18E-05 |
| chr11,1892551,1892608,+;p3@LSP1 | -3.8727 | -0.22264 | 3.82E-07 | 0.0002 |
| chr20,23807358,23807372,-;p1@CST2 | -4.43383 | 0.215653 | 8.76E-06 | 0.001576 |
| chr20,23860373,23860397,-;p1@CST5 | -4.5526 | -0.46037 | 5.31E-05 | 0.005591 |
| chr10,81320162,81320168,-;p1@SFTPA1,p1@SFTPA2 | -6.12028 | -0.01178 | 5.94E-05 | 0.00597 |
| chr5,180018490,180018505,-;p1@SCGB3A1 | -7.1244 | 0.761663 | 7.17E-06 | 0.001393 |
| chr2,85895315,85895331,-;p1@SFTPB | -7.20783 | 1.324048 | 6.29E-06 | 0.001285 |

Fold change (FC); counts per million reads (CPM); false discovery rate (FDR)

**Table S3 Gene ontology analysis**

| **Term** | **Count** | **%** | **p-value** | **Benjamini** | **DEGs** |
| --- | --- | --- | --- | --- | --- |
| Extracellular matrix organization | 16 | 9.4 | 2.40E-10 | 3.10E-07 | *F11R, APBB2, CCDC80,* ***COL1A1****, COL4A4, COL5A1, COL10A1,* ***COL11A1****,* ***ITGA11****, ICAM4, LAMA1, LOX, MFAP2, POSTN, SPP1, SPARC* |
| Cell adhesion | 17 | 9.9 | 3.60E-06 | 2.30E-03 | *CASS4, F11R, NUAK1, WISP1,* ***COL1A1****, COL5A1, DSG2, ISLR,* ***ITGA11****, ICAM4, IL32, LAMA1, LSAMP, LOXL2, POSTN, SPP1, ZYX* |
